# Influence of metallic particles and TNF on the transcriptional regulation of NLRP3 inflammasome-associated genes in human osteoblasts

**DOI:** 10.3389/fimmu.2024.1397432

**Published:** 2024-05-01

**Authors:** Marie-Luise Sellin, Doris Hansmann, Rainer Bader, Anika Jonitz-Heincke

**Affiliations:** Biomechanics and Implant Technology Research Laboratory, Department of Orthopedics, Rostock University Medical Center, Rostock, Germany

**Keywords:** NLRP3 inflammasome, human osteoblasts, metallic particles, osteolysis, joint replacement

## Abstract

**Introduction:**

The release of mature interleukin (IL-) 1β from osteoblasts in response to danger signals is tightly regulated by the nucleotide-binding oligomerization domain leucine-rich repeat and pyrin-containing protein 3 (NLRP3) inflammasome. These danger signals include wear products resulting from aseptic loosening of joint arthroplasty. However, inflammasome activation requires two different signals: a nuclear factor-kappa B (NF-κB)-activating priming signal and an actual inflammasome-activating signal. Since human osteoblasts react to wear particles via Toll-like receptors (TLR), particles may represent an inflammasome activator that can induce both signals.

**Methods:**

Temporal gene expression profiles of TLRs and associated intracellular signaling pathways were determined to investigate the period when human osteoblasts take up metallic wear particles after initial contact and initiate a molecular response. For this purpose, human osteoblasts were treated with metallic particles derived from cobalt-chromium alloy (CoCr), lipopolysaccharides (LPS), and tumor necrosis factor-alpha (TNF) alone or in combination for incubation times ranging from one hour to three days. Shortly after adding the particles, their uptake was observed by the change in cell morphology and spectral data.

**Results:**

Exposure of osteoblasts to particles alone increased NLRP3 inflammasome-associated genes. The response was not significantly enhanced when cells were treated with CoCr + LPS or CoCr + TNF, whereas inflammation markers were induced. Despite an increase in genes related to the NLRP3 inflammasome, the release of IL-1β was unaffected after contact with CoCr particles.

**Discussion:**

Although CoCr particles affect the expression of NLRP3 inflammasome-associated genes, a single stimulus was not sufficient to prime and activate the inflammasome. TNF was able to prime the NLRP3 inflammasome of human osteoblasts.

## 1 Introduction

After total joint replacement, wear-related osteolysis and aseptic loosening are the main risk factors of implant failure. Hereby, mechanical abrasion and chemical corrosion processes lead to the formation and release of wear products (1). Macrophages and osteoclasts are the main phagocytes that recognize the wear products, leading to activation and particle uptake (2). In response, the cells secrete various inflammatory mediators to recruit more immune cells (3). However, since wear particles can withstand enzymatic degradation, an intense immune response is mediated, resulting in a chronic foreign body reaction. The ongoing inflammatory response then affects the balance between bone formation and resorption, leading to osteolysis and loosening of the implant (4).

The potent proinflammatory cytokine Interleukin (IL)-1β is a driving factor of osteolysis by promoting osteoclast function and inhibiting osteoblast differentiation (4, 5). The release of mature IL-1β is tightly regulated by the nucleotide-binding oligomerization domain-leucine-rich repeat and pyrin-containing protein (NLRP)-3 inflammasome (4). The NLRP3 inflammasome is the best-described multimeric protein complex of inflammasomes and is a critical component of the innate immune system (6, 7). NLRP3 is a tripartite protein consisting of an amino-terminal pyrin domain, a central NOD, and an NLRP. An NLRP3 inflammasome is composed of an NLRP3 sensor protein, the adaptor apoptosis-associated Speck-like protein containing a caspase recruitment domain ([CARD] ASC), and a pro-caspase-1 (8). The oligomerized complex, which is concentrated in the cytoplasm, can recruit and cleave pro-caspase-1 via the CARDs of ASC, resulting in activated caspase-1 that can cleave pro-IL-1β, pro-IL-18, and gasdermin D into their mature forms (5, 9). The actual inflammasome-activating signal requires a prior priming signal that activates NF-κB, which, among other things, causes transcriptional upregulation of NLRP3 and pro-IL1β (6). This priming signal can be triggered by recognizing lipopolysaccharides (LPS) or IL-1β by pattern recognition receptors (7). Depending on the length of the priming stimulus, transcription-independent or transcription-dependent signaling pathways are initiated, which can lead to the activation of the inflammasome. While short exposures can lead to direct activation of the inflammasome, more prolonged stimuli result in *de novo* protein synthesis via transcriptional activation (10). Following priming, the inflammasome can be activated by various stimuli. The inflammasome activators do not interact directly with NLRP3 but instead induce a common cellular signal, which can be detected by NLRP3 (6). Various molecular and cellular stimuli, such as ion flux, mitochondrial dysfunction, production of reactive oxygen species (ROS) or lysosomal damage, as well as silica particles, asbestos crystals, and urate, ultimately activate the NLRP3 inflammasome (3, 5, 11–15). The NLRP3 inflammasome mediates the activation of caspase-1 (CASP1), IL-1β, and IL-18 to induce inflammation. However, overactivation of the inflammasome can also cause osteoblast dysfunction and induce pyroptosis (5, 16).

In recent years, the involvement of the inflammasome in macrophages has been shown to play an important role in the inflammatory response to wear particles (9, 17–19). Particle uptake by endocytosis leads to damage of the lysosomal membrane, which can trigger activation of the NLRP3 inflammasome. In addition, phagocytosis of particles leads to potassium efflux (20), which is required for inflammasome activation.

This study aimed to use short-term experiments to investigate the time required for human osteoblasts to take up metallic particles derived from cobalt-chromium alloy (CoCr) after initial contact and to initiate a molecular biological response. Since it has already been shown that osteoblasts can form the NLRP3 inflammasome (21, 22), the question arose to which extent particles have an impact on the inflammasome. Moreover, it is highly interesting whether particles can prime independently or whether a separate priming signal is required. As the amount of lipopolysaccharides (LPS) present in aseptic loosening is controversial (4, 23, 24), Jämsen et al. (2020) identified tumor necrosis factor alpha (TNF) as a priming signal that induces the inflammasome in the presence of particles. Although osteoblasts are among the cells that come into contact with wear products at a very early stage (1), the mechanisms underlying the initial contact between particles and cells are still unknown (25). Therefore, time-dependent gene expression profiles were generated to investigate the response of human osteoblasts to different priming stimuli and their influence of the transcriptional regulation of NLRP3 inflammasome-associated genes. Elucidating the uptake mechanisms of wear particles is a key factor in determining the effect of the particles assessing their subsequent fate and toxic potential (1, 26).

## 2 Materials and methods

**Figure 1:**
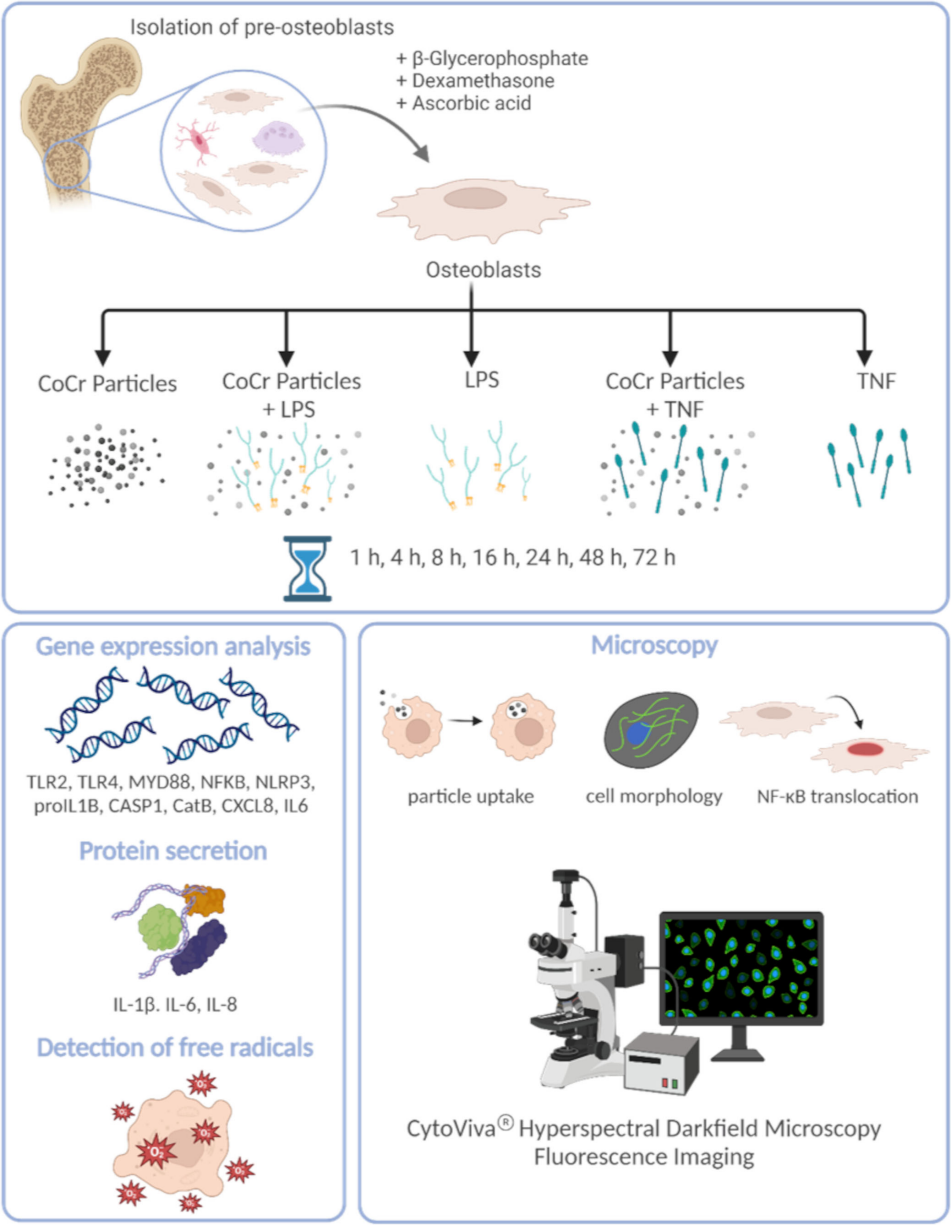
Schematic overview of the experimental setup and the methods used. The figure was created on 10/18/2023 at 10:55 AM with Biorender (Agreement number: YG26QJZVPQ) (https://biorender.com).

A schematic overview of the experimental setup and the methods used is shown in **Figure 1**.

### 2.1 Cell culture of human primary osteoblasts

To realistically illustrate disease-relevant processes in aseptic loosening, the isolation of human primary osteoblasts was performed according to a well-established protocol by Lochner et al. (27) from femoral heads of patients with osteoarthritis (OA). The femoral heads were provided after obtaining informed consent from patients undergoing total hip arthroplasty. The study was approved by the local ethical committee, ref. number A 2010–0010. A total of 21 patients were recruited.

The isolated cells were cultured under standard cell culture conditions at 37°C and 5% CO_2_ at 95% humidity in calcium-free Dulbecco’s Modified Eagle Medium (DMEM; Pan-biotech, Aidenbach, Germany) containing 10% fetal calf serum (FCS; PAN-Biotech, Aidenbach, Germany), 1% amphotericin B, 1% penicillin-streptomycin, and 1% 2-(4-(2-hydroxyethyl)-1-piperazinyl)-ethanesulfonic acid (HEPES buffer; all: Sigma-Aldrich, Munich, Germany). The absence of calcium in the cell culture medium suppresses the mineralization of the osteoblastic cells. To maintain the osteogenic phenotype, 10 mM β-glycerophosphate, 50 μg/mL ascorbic acid, and 100 nM dexamethasone were added to the cell culture medium (all: Sigma-Aldrich, Munich, Germany).

For cell culture experiments, cells from passage 3 were seeded in 12 well cell culture plates with 30 000 cells per well and cultured for 24 h to allow cell adherence. Additionally, for staining experiments, 20 000 osteoblasts were seeded in 8 well chamber slides (ibidi GmbH, Gräfelfing, Germany) and cultured for 24 h.

### 2.2 Exposure of osteoblasts to metallic particles and TNF

Osteoblasts were treated with 0.01 mg/mL cobalt-chromium-molybdenum (CoCr) particles with an average size of 0.5 µm, 0.05 µg/mL TNF (abcam^®^, UK) or with a combination of CoCr and TNF. Untreated cells served as negative controls, and cells stimulated with 1 µg/mL lipopolysaccharides (LPS; Sigma-Aldrich, Munich, Germany) and CoCr + LPS served as positive controls (Data shown in **Supplementary Material**). Osteoblasts were exposed over 10 min, 15 min, 20 min, 25 min, 30 min, 45 min, 1 h, 4 h, 8 h, 16 h, 24 h, 48 h, or 72 h.

### 2.3 Determination of cell morphology

Actin staining with diamidino-2-phenylindole dihydrochloride (DAPI) counterstain was used to analyze changes in the structure of the cells’ cytoskeleton after particle exposure. For this purpose, the medium was removed, and the cells were washed with phosphate-buffered saline (PBS; biochrom, Berlin, Germany). The following steps were performed protected from light and at room temperature. Before staining, cells were fixed with 4% paraformaldehyde (PFA; pH: 7.0). After 10 min, cells were rinsed with PBS for 30 seconds. Then, the cell membrane was permeabilized by adding a permeabilization buffer containing 0.05% Triton-X (Merck KGaA, Darmstadt, Germany) for 5 min. Osteoblasts were rewashed with PBS for 30 seconds before 100 nM actin staining solution (100 nM Acti-Stain 488 Fluorescent Phalloidin, Cytoskeleton, Denver, CO, USA) was added to the cells for 30 min. After washing three times with PBS, osteoblasts were incubated with DAPI (Merck KGaA, Darmstadt, Germany) for 5 min to counterstain the nuclei of the cells. The staining solution was removed, and cells were washed with PBS and stored at 4°C until microscopic examination.

### 2.4 NF-κB translocation

Staining was used to assess the translocation of NF-κB. For this purpose, osteoblasts were stimulated for 5 min, 10 min, 15 min, 20 min, 25 min, 30 min, 1 h, 4 h, 8 h, 16 h, 24 h, 48 h, and 72 h with CoCr particles, LPS, TNF, CoCr particles + LPS (CoCr + LPS) or CoCr particles + TNF (CoCr + TNF). For each time point, untreated cells were included as negative controls, and osteoblasts treated with 50 ng/mL TNF (ab9642, abcam^®^, UK) for 30 min served as positive controls. After each stimulation period, the medium was removed, and the cells were washed with PBS and fixed with 4% PFA for 10 min. After washing with PBS, the cells were blocked and permeabilized with 5% goat serum (abcam^®^, UK) and 0.3% Triton-X-100 (Merck KGaA, Darmstadt, Germany) for 1 h at room temperature. Subsequently, overnight NF-κB staining was performed with an anti-NF-κB rabbit anti-human antibody (NF-κB p65 (C22B4), Cell Signaling Technology^®^, Cambridge, UK) at a dilution of 1:100 and 1% goat serum at 4°C. The following steps were performed under light protection. The primary antibody was removed, and the cells were washed with PBS. The secondary antibody Alexa Fluor 594 goat anti-rabbit was added at a dilution of 1:500 and 1% goat serum. To verify the specific binding of the antibody, a negative control was included; the cells were incubated without the primary antibody and then the secondary antibody was added. No non-specific binding of the antibody was detected. After 2 h, the staining solution was removed, the cytoskeleton of the cells was stained with phalloidin (actin) stain, and the nuclei were stained with DAPI as described above. Afterward, the cells were embedded and stored at 4°C until microscopic evaluation.

### 2.5 ASC-speck formation

The formation and activation of the NLRP3 inflammasome in osteoblasts was investigated by staining ASC specks. For this purpose, osteoblasts were stimulated for 8 h with CoCr particles, LPS, TNF, CoCr + LPS or CoCr + TNF. For each time point, untreated cells were included as negative controls, and osteoblasts treated with CoCr, LPS, or TNF and 5 mM ATP (tlrl-atpl, InvivoGen, San Diego, CA, USA) for 45 min served as positive controls. After stimulation, the medium was removed, and the cells were washed with PBS. Osteoblasts were than fixed with 4% PFA for 10 min. After washing with PBS three times, cells were blocked and permeabilized with UltraCruz^®^ Blocking Reagent (sc-516214; Santa Cruz Biotechnology, Santa Cruz, CA, USA) and 0.3% Triton-X-100 (Merck KGaA, Darmstadt, Germany) for 30 min at room temperature. After washing with PBS, overnight ASC staining was performed using ASC/TMS1/PYCARD (B-3) Alexa Fluor^®^ 488 (sc-514414; Santa Cruz Biotechnology, Santa Cruz, CA, USA) at a dilution of 1:50 and 1.5% Blocking Reagent at 4°C. Negative control staining with a fluorescence-conjugated goat anti-rabbit IgG antibody (Sigma-Aldrich, Munich, Germany) was included to test the specific binding of the antibody. Nonspecific binding was not detected. After that, the staining solution was removed and the nuclei were stained with DAPI as described above. The microscopic examinations were performed using a Keyence BZ-X810 microscope (Keyence Germany GmbH, Neu-Isenburg, Germany) with a 20x and a 40x objective.

### 2.6 Microscopic examinations

Microscopic examinations were performed using the CytoViva^®^ Enhanced Darkfield Hyperspectral Microscope System (CytoViva, Inc., Auburn, AL, USA) and a 60 x oil objective. CytoViva’s dual-mode fluorescence module allows simultaneous real-time observation of fluorescent and non-fluorescent sample components. The green fluorescent cytoskeleton of the cells was imaged at a wavelength of 525 nm using a bandpass emission filter (69002m, Chroma Technology Corporation, VT, USA). The blue fluorescence of the nuclei stained with DAPI was recorded at a wavelength of 461 nm. The red fluorescence of the NF-κB signal was detected at a wavelength of 625 nm. Image acquisition was performed using *Ocular Imaging* software (Teledyne Photometrics, Tucson, AZ, USA). In addition, images were acquired in the darkfield to visualize unstained structures such as intracellular vesicles, large endosomes, and granules.

### 2.7 Hyperspectral imaging

Hyperspectral images and data were captured using an optical microscope (Olympus BX 41) equipped with an advanced darkfield illumination system and integrated hyperspectral imaging (HSI) spectrometer (CytoViva Inc., Auburn, AL, USA). Spatial and spectral data (wavelengths between 400 nm and 1000 nm) were collected at 60 x magnification. Detection of particles occurred when the signal for the material in the given pixel was more significant than the background noise. Spectral libraries were created by analyzing solutions containing CoCr particles in DMEM medium. To create a spectral library, pixels that could be identified as particles were selected and marked using the region of interest (ROI) tool. The ROI was converted into a spectral library. After hyperspectral imaging of particles in solution, cells treated with CoCr particles were imaged. Hyperspectral analysis was performed using Environment for Visualization (ENVI) v4.8 software (Exelis Visual Information Solutions, Boulder, CO, USA).

### 2.8 Gene expression analysis

Following the manufacturer’s protocol, total ribonucleic acid (RNA) was isolated using the innuPREP RNA Mini Kit 2.0 (Analytik Jena GmbH, Jena, Germany). After elution of the RNA into a fresh sterile tube using RNase-free water, RNA concentration was measured using the Tecan Infinite^®^ 200 microplate reader and the NanoQuant Plate™ (both: Tecan Group AG, Maennedorf, Switzerland). RNase-free water served as blank. Subsequently, the High Capacity cDNA Reverse Transcription Kit (Applied Biosystems, Forster City, CA, USA) was used to transcribe 50 ng of RNA from each sample into complementary deoxyribonucleic acid (cDNA). The PCR was done with the following protocol: 10 min at 25°C, 120 min at 37°C, and 5 min at 85°C in a thermocycler (Analytik Jena GmbH, Jena, Germany). Afterward, samples were diluted in 20 µl RNase-free water and stored at – 20°C.

Expression levels of recognition-, inflammasome- and inflammation-associated genes were determined by a semi-quantitative real-time polymerase chain reaction (qPCR; qTower 2.0, Analytik Jena GmbH, Jena, Germany) using the innuMIX qPCR MasterMix SyGreen (Analytik Jena AG, Jena, Germany) and the primer pairs listed in **Table 1**. Each sample was measured in duplicate. QPCR was performed following the protocol: 2 min at 95°C, followed by 40 times of rotation of denaturation of 5 sec at 95°C, and annealing/elongation for 25 sec at 60-65°C. The relative amount of each mRNA compared with the housekeeping gene β-Actin was calculated by the equation ΔCt = Ct_target_-Ct_housekeeping gene_. The relative expression of target mRNA of unstimulated and treated cells was calculated using the 2(^-ΔΔCt^) method (relative to unstimulated control).

**Table 1:** cDNA target sequences for semi-quantitative real-time PCR.

Gene	Forward primer (5’ – 3’)	Reverse Primer (5’ – 3’)
**Activator protein-**1 (*AP-1*)	GTGCCGAAAAAGGAAGCTGG	CTGCGTTAGCATGAGTTGGC
**Cathepsin B** (*CATB*)	AAGCCACCCCAGAGAGTTATG	ACCATTACAGCCAGCAGGAG
**Caspase 1** (*CASP1*)	GAAAAGCCATGGCCGACAAG	AAATTTGGCATGCCTGTGCC
**Hypoxia Inducible Factor 1 Subunit Alpha** (*HIF1A*)	GTACCCTAACTAGCCGAGGAAGAA	GTGAATGTGGCCTGTGCAGT
**Interleukin 6** (*IL6*)	TGGATTCAATGAGGAGACTTGCC	CTGGCATTTGTGGTTGGGTC
**Interleukin 8** (*CXCL8*)	TCTGTGTGAAGGTGCAGTTTTG	ATTTCTGTGTTGGCGCAGTG
**Myeloid differentiation primary response 88** (*MYD88*)	GATGATTACCTGCAGAGCAAGG	TCTGATGGGCACCTGGAGAG
**Nuclear Factor of kappa B** *(NFκB)*	CCATATTTGGGAAGGCCTGAAC	TGAAGGTATGGGCCATCTGTTG
**NLR family pyrin domain containing 3** (*NLRP3*)	AGGAGAACTTTCTGTGTGGACC	TTCTCTGTCTGACCCCTCGG
**Pro-Interleukin 1β** (*pro-IL1B*)	TACTCACTTAAAGCCCGCCT	ATGTGGGAGCGAATGACAGA
**Toll-like receptor 2** (*TLR2*)	GGAGTTCTCCCAGTGTTTGGT	TTCCTGCCTTCACTTGGTCA
**Toll-like receptor 4** (*TLR4*)	GGTCAGACGGTGATAGCGAG	TTTAGGGCCAAGTCTCCACG
**Tumor necrosis factor alpha** (*TNFA*)	GTTGTAGCAAACCCTCAAGCTG	GAGGTACAGGCCCTCTGATG
**β-Actin**	CTTCCTGGGCATGGAGTC	AGCACTGTGTTGGCGTACAG

Primer pairs were purchased from Merck KGaA (Darmstadt, Germany).

### 2.9 Quantification of intracellular and secreted proteins

Protein levels of IL-1β, IL-6, and IL-8 (Human IL-1-beta ELISA Ready-SET-Go!™, Human IL-6 ELISA Ready-SET-Go!™, Human IL-8 ELISA Ready-SET-Go!™, ThermoFisher Scientific, Waltham, MA, USA) were determined in the supernatant of control and exposed osteoblasts via enzyme-linked immunosorbent assay. For this purpose, supernatants at the respective time points were collected and stored at -20°C before quantification. ELISAs were performed according to the manufacturer’s recommendations. Absorbance was measured at 405 nm (reference wavelength: 630 nm; IL-6: 570 nm) using the Tecan Infinite^®^ 200 Pro microplate reader (Tecan Group AG, Maennedorf, Switzerland). The sample concentrations were calculated using a standard curve. Normalization of protein content to total protein was performed using the Qubit Protein Assay Kit and Qubit 1.0 (both: Thermo Fisher Scientific, Waltham, MA, USA) according to the manufacturer’s instructions.

To determine intracellular IL-1β levels, osteoblasts were treated with CoCr, LPS, TNF, CoCr + LPS, or CoCr + TNF for 8 h, 24 h, and 72 h, respectively. After incubation, cells were lysed with cell lysis buffer II (Thermo Fisher Scientific, Waltham, MA, USA) according to the manufacturer’s instructions. Subsequently, protein concentrations were determined by ELISA (Human IL-1-beta ELISA Ready-SET-Go!™, ThermoFisher Scientific, Waltham, MA, USA) as described above. Normalization of protein content to intracellular protein was performed using the Qubit Protein Assay Kit and Qubit 1.0 (both: Thermo Fisher Scientific, Waltham, MA, USA) according to the manufacturer’s instructions.

### 2.10 Detection of free radicals

Total free radical concentrations in supernatants and lysed osteoblasts after 8 h and 24 h of treatment were determined using the OxiSelect™ *In Vitro* ROS/RNS Assay Kit (cell biolabs, inc., San Diego, CA, USA). The assay was performed according to the manufacturer’s instructions. A standard curve of fluorescent 2’, 7’-dichlorodihydrofluorescein (DCF) was used to determine the concentration of the free radicals (in nM).

### 2.11 LDH assay

To quantify the induction of pyroptosis as a marker of inflammasome activation, a lactate dehydrogenase (LDH) activity assay (Sigma-Aldrich, Munich, Germany) was performed. For this assay, 10 000 cells per well were seeded in quadruplicate in a 96-well plate and incubated at 37°C for 24 hours. The cells were then treated with CoCr, LPS, TNF, CoCr + LPS and CoCr + LPS for 8 h and 72 h, respectively. In addition, ATP-activated samples were included as positive samples and untreated samples as negative controls. After the incubation period, 50 µl of the supernatant was removed and transferred to a new 96-well plate. The assay was performed according to the manufacturer’s instructions. The absorbance of the samples was measured at 450 nm in a microplate reader (Tecan Group AG, Maennedorf, Switzerland).

### 2.12 Graphical illustration and statistics

All experiments were performed with osteoblasts from 21 individual donors (male: n=10, mean age: 74 ± 7.8 years; female: n=11, mean age: 73 ± 6.7 years). Data were presented as individual values (one data point per donor) with median and interquartile ranges or heatmaps with median, and statistical analysis was performed using GraphPad Prism, version 8.0 (GraphPad Software, San Diego, CA, USA). If not otherwise stated, different stimulation groups were compared using repeated measures two-way ANOVA with Bonferroni’s multiple comparison *post hoc* test, as required. A p-value of less than 0.05 was defined as statistically significant. To improve the clarity of the figures, significant differences within a treatment between time points have not been shown in the graphs.

### 2.13 Data availability

The datasets generated and/or analyzed during the current study are available in the Zenodo repository and are available from the corresponding author upon reasonable request.

## 3 Results

### 3.1 Influence of particle exposure on osteoblastic morphology and hyperspectral data

**Figure 2:**
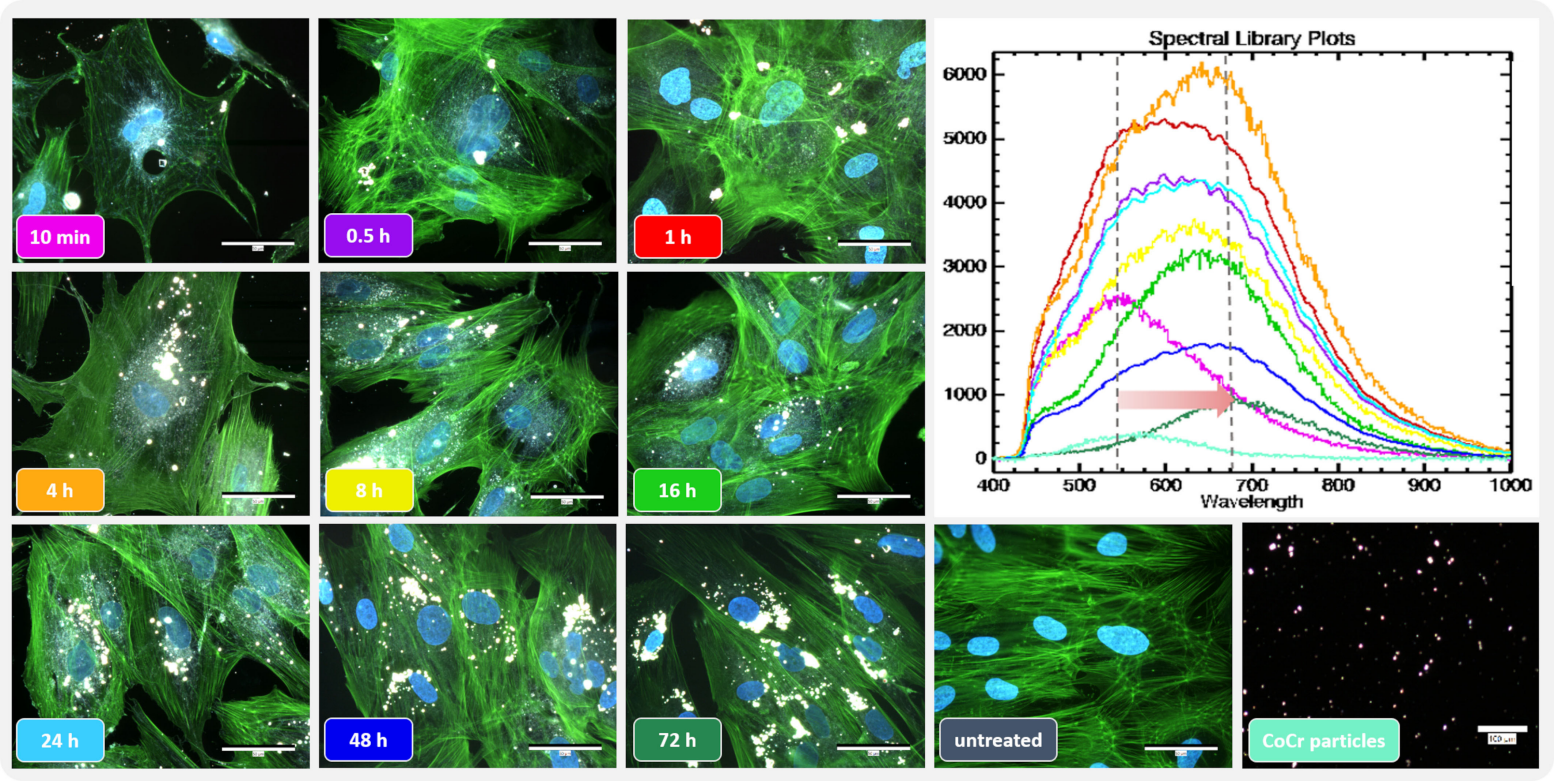
Influence of short-term particle exposure on cell morphology of human osteoblasts and influence of particle incorporation on hyperspectral data. (Left) Combined fluorescence darkfield images of osteoblasts exposed to CoCr particles at different time points were taken with the CytoViva^®^ microscope system. The actin cytoskeleton of cells was stained with phalloidin (green fluorescence), and the nuclei were counterstained with DAPI (blue fluorescence). (Top right) To determine particle uptake, hyperspectral images (HSI) of CoCr particles in cell culture medium and of particles after incubation with cells were taken with the CytoViva^®^ microscope system, and the mean spectral profiles are shown in a spectral library plot. Bar: 50 µm; 100 µm (CoCr particles).

The actin cytoskeleton and nucleus of the cells were stained to assess the influence of the metal particles on osteoblast morphology. Darkfield fluorescence images were taken to detect the distribution of particles that appeared brightly shining (**Figure 2** left). Unstimulated osteoblasts showed elongated bodies with long, ordered actin structures with strong interconnectivity (**Figure 2**, untreated). In comparison, the structure changed after particle exposure. The most remarkable morphological change was observed within the first hour after particle addition, with cells appearing more unstructured, smaller, and rounder with shortened actin filaments compared to unstimulated cells. Shortly after treatment, most of the particles were localized at the borders of the cells, whereas over time, an accumulation of particles was observed centrally, close to the nucleus. Furthermore, the morphology returned to the structure of untreated cells over time.

The uptake of the particles was assessed using the hyperspectral data (HSI; **Figure 2** top right). After adding the particles, their hyperspectral data show a high similarity to the particles recorded in cell culture medium only (**Figure 2** Spectral library plot: turquoise line). At later time points, a shift in the spectral peaks towards higher wavelengths was observed. In addition, the intensity of the signal initially increased up to 4 h particle exposure as the accumulation of particles led to an increase in the signal. However, this intensity decreased as the later time points were recorded.

### 3.2 Biological response of human osteoblasts following CoCr and TNF exposure

#### 3.2.1 Gene expression of TLR signaling

**Figure 3:**
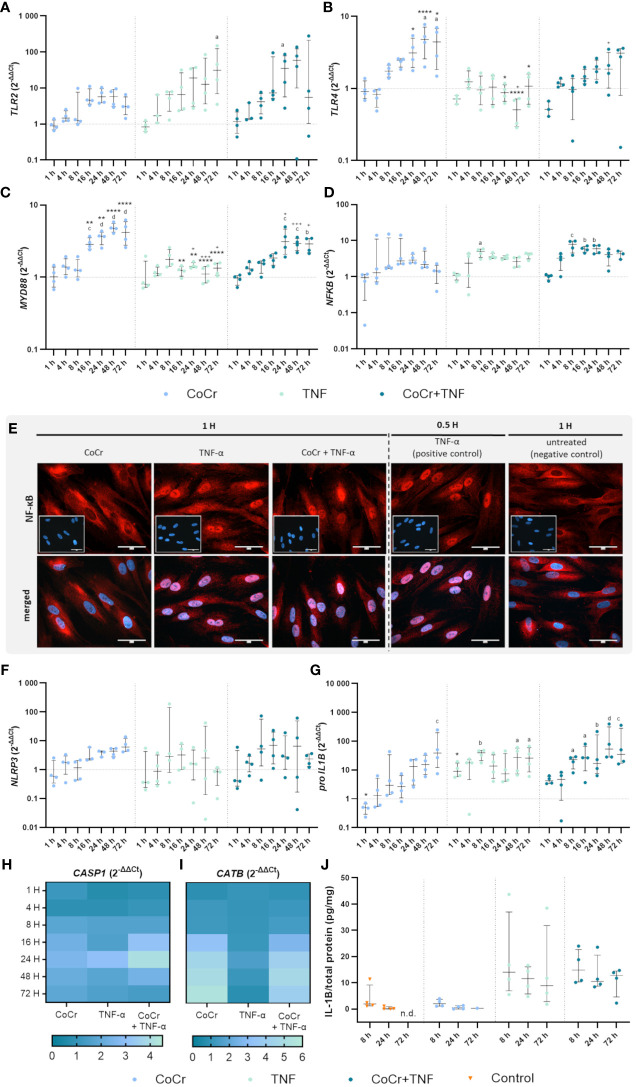
Gene expression analysis of markers of TLR signaling **(A-D)** and NLRP3 inflammasome **(F-J)** in human osteoblasts treated with CoCr (blue dots), TNF (light turquoise dots), or CoCr + TNF (dark blue dots) compared to the untreated control (J: orange triangle) and evaluation of translocation of NF-κB to the nucleus via NF-κB staining **(E)**. **(A-D, F-I)** The total RNA of osteoblasts was isolated, and relevant genes were examined via qPCR. The results were calculated by the 2^-ΔΔCt^ method and normalized to the unstimulated control (dotted line; in heatmaps: 1). **(J)** Intracellular IL-1β protein of cell lysates lysates after 8 h, 24 h, and 72 h of stimulation was examined by ELISA and related to the total protein amount. The results are shown as individual values (one data point per donor) with median and interquartile ranges **(A-D, F, G, J)** or as median within the heatmaps (**H**, **I**,; n=4). Statistical significance was determined using the 2-way ANOVA and Bonferroni multiple comparison *post hoc* test **(A-I)** and Kruskal-Wallis test with Dunn’s multiple comparisons test **(J)**: *p<0.05; **p<0.01; ****p<0.0001 (significance between single stimulations); ^+^p<0.05; ^++^p<0.01; ^+++^p<0.001 (significance between TNF and CoCr + TNF); ^a^p<0.05; ^b^p<0.01; ^c^p<0.001; ^d^p<0.0001 (significance to unstimulated control). Significant differences within a treatment between time points are not shown in the graphs. n.d., not detectable.

**Figure 4:**
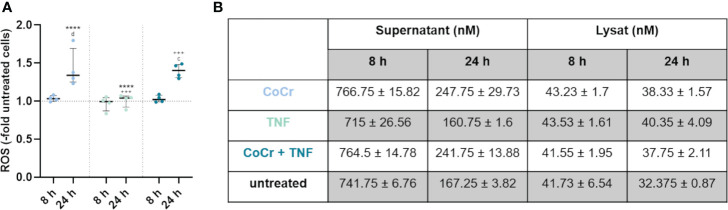
Total free radical concentration from the supernatant and lysed cells after 8 h and 24 h treatment with CoCr particles (blue dots), TNF (turquoise dots), or CoCr + TNF (dark blue dots) of cells relative to untreated control. Statistical significance was determined using the 2-way ANOVA and Bonferroni multiple comparison *post hoc* test **(A)**: ****p<0.0001 (significance between single stimulations); ^+++^p<0.001 (significance between LPS and CoCr + LPS); ^c^p<0.001, ^d^p<0.0001 (significance to unstimulated control). Significant differences within a treatment between time points are listed in the text and not shown in the graphs. **(B)** Free radical concentration in the supernatant and from lysed cells after 8 h and 24 h. Data in the table are presented as mean values ± SEM (n=4, one data point per donor).

**Figure 5:**
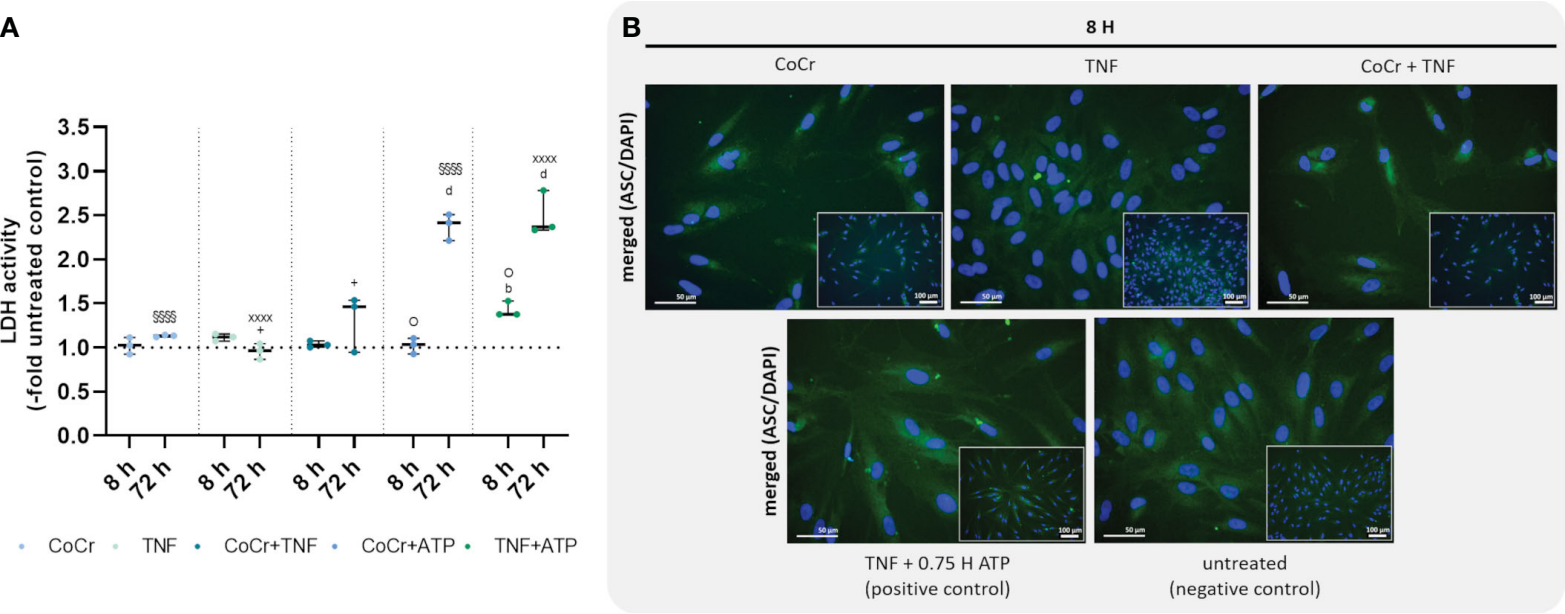
Effects on lactate dehydrogenase (LDH) activity **(A)** and staining of ASC-speck formation **(B)**. LDH activity was measured in supernatants of osteoblasts after exposure with CoCr (blue dots), TNF (light turquoise dots), CoCr+TNF (dark blue dots), CoCr+ATP (dark blue dots), and TNF+ATP (green dots). The results are shown as individual values (one data point per donor) with median and interquartile ranges (n=3). Statistical significance was determined using the 2-way ANOVA and Bonferroni multiple comparison *post hoc* test: ^+^p<0.05 (significance between TNF and CoCr + TNF); ^b^p<0.01; ^d^p<0.0001 (significance to unstimulated control); ^§§§§^p<0.0001 (significance between CoCr and CoCr + ATP); ^XXXX^p<0.0001 (significance between TNF and TNF + ATP); ^O^p<0.05 (significance between CoCr + ATP and TNF + ATP). An ASC (green) staining was used to investigate the formation of NLRP3 inflammasome after stimulating osteoblasts with CoCr particles, TNF, and CoCr + TNF for 8 h. Unstimulated cells were included as negative controls. Osteoblasts treated with TNF and ATP served as positive control. The nuclei were stained with DAPI (blue). Bars: 50 µm; 100 µm.

To investigate the extent to which human osteoblasts recognize CoCr particles and which intracellular signaling cascade is thereby initiated, corresponding gene expression studies were performed at defined time points between 1 h and 72 h. First, the response of human osteoblasts to LPS was investigated since LPS, as a TLR4 agonist, is a known stimulus for priming the inflammasome (**Supplementary Figures S1**, **S2**). Since LPS had only a minor effect on the expression of genes associated with TLR signaling, the observed effects of the combination treatment CoCr + LPS were instead attributed to the influence of the particles (**Supplementary Figures S1A–C**). Only the expression of *NF-κB* was significantly upregulated after four hours of LPS treatment compared to CoCr treatment (p=0.0027) (**Supplementary Figure S1D**). Based on these findings, TNF was analyzed as an alternative priming stimulus for osteoblasts (**Figures 3**–**5**).

An increased *TLR2* expression level after CoCr treatment was detected after 16 hours, which remained elevated after 24, 48, and 72 h compared to untreated cells (**Figure 3A**). CoCr increased the expression of *TLR4* after 8 h, with significantly enhanced expression levels after 48 h (p=0.01) and 72 h (p=0.0424) compared to untreated cells (**Figure 3B**). *Myeloid differentiation primary response 88* (*MyD88*) showed significantly increased mRNA levels at 16 h (p=0.0006), 24 h (p<0.0001), 48 h (p<0.0001), and 72 h (p<0.0001) following particle exposure compared to untreated cells (**Figure 3C**). The gene expression of the *nuclear factor of kappa B* (*NF-κB*) showed a slight increase after treatment of cells with CoCr, peaking at 24 h, followed by a decrease in expression (**Figure 3D**).

Over time, treating osteoblasts with TNF resulted in a slow increase in *TLR2* gene expression (**Figure 3A**). Stimulation for 72 h resulted in a significant upregulation of mRNA levels compared to control (p=0.0119). Exposure of osteoblasts to CoCr + TNF increased *TLR2* expression. Treatment over 24 h resulted in significantly higher expression rates than the untreated control (p=0.0248). Treatment of osteoblasts with TNF did not change *TLR4* expression compared to untreated cells (**Figure 3B**). Compared to CoCr treatment, *TLR4* expression was significantly reduced after 24 h (p=0.0184), 48 h (p<0.0001), and 72 h (p=0.0137). Similarly, a significantly downregulated expression was observed after 48 h of TNF treatment compared to treatment with CoCr + TNF (p=0.0111). Gene expression following stimulation with CoCr + TNF showed a similar trend as the CoCr treatment. A slight delay from 16 h onwards was detectable after CoCr exposure. Osteoblasts treated with TNF alone did not show significantly altered *MyD88* gene expression levels compared to untreated cells (**Figure 3C**). However, at 16 h (p=0.0042), 24 h (p=0.0036), 48 h (p<0.0001), and 72 h (p<0.0001), TNF treatment resulted in significantly lower mRNA levels compared to osteoblasts exposed to CoCr particles. TNF stimulation alone decreased expression compared to CoCr + TNF (p_24h_=0.00221; p_48h_=0.001; p_72h_=0.0209). Further, CoCr + TNF upregulated *MyD88* in osteoblasts after 4 h, and the mRNA levels were significantly increased after 24 h (p=0.0002), 48 h (p=0.0007), and 72 h (p=0.0012) compared to untreated cells. The gene expression of *NF-κB* was upregulated after TNF treatment, with significantly enhanced values after 8 h (p=0.0157) compared to the control cells (**Figure 3D**). The expression level after TNF treatment remained slightly elevated for the time points studied. CoCr + TNF stimulation resulted in significantly increased expression rates after 8 h, 16 h, and 24 h compared to control (p_8h_=0.0005; p_16h_=0.0024; p_24h_=0.0025).

#### 3.2.2 Microscopic examination of NF-κB translocation

The translocation of the transcription factor NF-κB into the nucleus was investigated by immunocytochemistry. NF-κB was mainly observed in the cytoplasm of untreated cells. Osteoblasts treated with TNF for 30 min were included as a positive control. If the nuclei stained red, it was assumed that translocation of NF-κB into the nucleus occurred. As the cells already showed translocation after one hour of LPS treatment (**Supplementary Figure S1E**), other earlier time points were examined. The induction of the reaction could already be detected after 25 minutes of LPS treatment (**Supplementary Figure S6**), but the best signal intensity was observed after 1 h. After one hour of TNF treatment, a clear translocation of NF-κB into the nucleus was observed (**Figure 3E**), whereas osteoblasts exposed to CoCr showed no nuclear staining. The intensity of the staining decreased with time, so only example images for 1 hour are shown. However, the reaction was studied for all the time points described, and the images for the other time points can be found in the Supplementary (**Supplementary Figures S3–5**). In further experiments, earlier time points were investigated to determine when osteoblasts initiate translocation in response to CoCr, TNF, or CoCr + TNF. TNF treatment for 10 min was sufficient to induce the response in osteoblasts (**Supplementary Figure S7**). In contrast, since no translocation was observed in response to CoCr particles, the induction of the NF-κB pathway in the combination treatment with CoCr + TNF was due to the influence of TNF. A decrease in signal intensity was observed for an incubation time of 4 h with TNF. After 8 h of incubation, no translocation within the nucleus was detectable (**Supplementary Figure S8**).

#### 3.2.3 Gene expression analysis of inflammasome signaling

Osteoblasts exposed to CoCr showed a slightly increased expression of *NLRP3* after 16 h of treatment (**Figure 3F**) and a slight increase in *CASP1* mRNA levels from 8 h particle exposure compared to untreated osteoblasts (**Figure 3H**). In comparison, LPS stimulation did not alter *NLRP3* gene expression, but CoCr + LPS tended to increase gene expression between 16 h and 48 h (**Supplementary Figure S1F**). Although *NLRP3* gene expression revealed strong donor-dependent differences following exposure either to TNF or CoCr + TNF, it was also observed that the expression profile of both treatments was very similar (**Figure 3F**). Exposure of osteoblasts to TNF or CoCr + TNF resulted in a slight increase in *CASP1* gene expression after 8 h. The combined stimulation with CoCr + TNF also led to a peak in *CASP1* gene expression after 24 h (**Figure 3H**).

*Cathepsin B* (*CATB*) expression showed a similar profile after CoCr, CoCr + LPS, and CoCr + TNF treatment, with mRNA levels increasing from 16 h treatment onwards. Neither LPS (**Supplementary Figure S1I**) nor TNF (**Figure 3I**) alters the gene expression profiles compared to untreated cells.

*Pro-IL1B* expression (**Figure 3G**) increased continuously over the observation period after exposure to CoCr particles with significantly enhanced mRNA transcripts after 72 h (p=0.001 compared to control). LPS stimulation resulted in a significant increase in *pro-IL1B* expression compared to CoCr exposure (p_4h_<0.0001), (p_8h_ =0.0114), (p_16h_ =0.0313), and (p_24h_ =0.0125) (**Supplementary Figure S1G**). Further, CoCr + LPS led to significantly enhanced mRNA transcripts compared to CoCr treatment after 4 h (p=0.0079, **Supplementary Figure S1G**). After 1 h of TNF stimulation, *pro-IL1B* mRNA was increased compared to the control (**Figure 3G**). This was also evident compared to CoCr exposure after 1 h (p=0.0172). Osteoblasts treated with TNF showed a continuously increased expression of *pro-IL1B*, with significant differences after 8 h (p=0.0020), 48 h (p=0.0173), and 72 h (p=0.0127). Co-stimulation of cells with CoCr + TNF led to upregulated mRNA levels as early as 1 h, increasing over time. An increase in the expression of *pro-IL1B* was observed over time, and significantly increased gene expression was determined for 8 h (p=0.0245), 16 h (p=0.0143), 24 h (p=0.0082), 48 h (p<0.0001), 72 h (p=0.0004).

While treatment with LPS and CoCr + LPS resulted in an increased concentration of intracellular IL-1β (**Supplementary Figure S1J**), treatment with CoCr did not alter the concentration compared to untreated osteoblasts (**Figure 3J**). Also, intracellular IL-1β levels (**Figure 3J**) were increased after treatment of osteoblasts with TNF and CoCr + TNF compared to CoCr alone. Further, a slight decrease in IL-1β was observed over time.

#### 3.2.4 Detection of free radicals

Measurement of free radicals in the supernatants and lysed cells (**Figure 4A**) showed a significant increase in production after 24 h following particle treatment compared to 8 h (p<0.0001), untreated osteoblasts (p<0.0001), and LPS (p<0.0001) (**Supplementary Figure S2A**). Further, treatment of osteoblasts with CoCr + LPS resulted in increased free radical production after 24 h, which was significantly increased compared to treatment for 8 h (p<0.0001), untreated control (p<0.0001), and LPS treatment alone (p<0.0001). After 24 h treatment with TNF measurement of free radicals in the supernatants and lysed cells (**Figure 4A**) showed a significantly decreased concentration compared to particle exposure (p<0.0001). Treatment of osteoblasts with CoCr + TNF also resulted in increased free radical production after 24 h, which was significantly increased compared to 8 h treatment (p<0.0001), untreated control (p=0.0001), and TNF treatment alone (p=0.0002).

Looking at the individual values (**Figure 4B**, **Supplementary Figure S2B**), it is visible that an incubation time of 8 h resulted in a higher concentration of free radicals compared to 24 h. Furthermore, the concentration of free radicals is higher in the supernatants than in the lysates. The concentration of free radicals appeared relatively constant within the cell, whereas it decreased in the supernatants with increasing time.

#### 3.2.5 LDH activity and microscopic examination of ASC speck formation

An LDH activity assay was performed to quantify pyroptosis and inflammasome activation. Osteoblasts treated with CoCr, TNF, or CoCr + TNF for 8 h showed no change in LDH activity compared to the control (**Figure 5A**). Cells treated with TNF for 8 h and subsequently activated with ATP showed a significant increase in LDH activity compared to control (p=0.0037) and CoCr-treated and ATP-activated cells (p=0.0124). After 72 h, osteoblasts that were treated with CoCr or TNF, and ATP-activated showed significantly higher LDH activity compared to untreated control and non-activated samples (all: p<0.0001). At 72 h, increased LDH activity was also detected after CoCr + TNF treatment compared to TNF treatment (p=0.037). Activation of LPS-treated osteoblasts also resulted in a significant increase in LDH activity (all: p<0.0001). The combined treatment of cells with CoCr + LPS resulted in a significant increase at 72 h compared to untreated controls (p<0.0001), CoCr (p=0.0011) and LPS (p<0.0001) (**Supplementary Figure S9A**).

To investigate whether a single priming signal is sufficient to form the NLRP3 inflammasome, speck formation was visualized by ASC staining after 8 h (**Figure 5B**). After CoCr and CoCr + TNF treatment, only the diffuse cytoplasmic staining was observed, indicating that the inflammasome was not activated. Treatment of osteoblasts with TNF resulted in the formation of sporadic ASC specks. Although an increase in LDH activity was detected in TNF-treated and LPS-treated and ATP-activated samples, only a few perinuclear or extracellular ASC specks were detected (**Figure 5B**, **Supplementary Figure S9B**).

#### 3.2.6 Gene expression and protein secretion of inflammation markers

**Figure 6:**
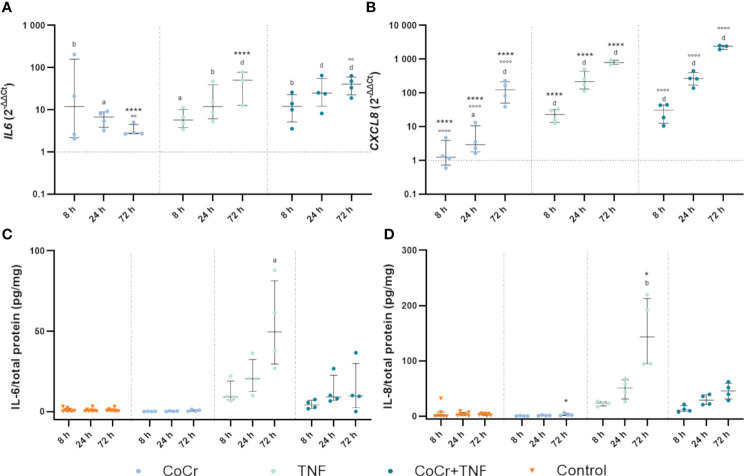
Markers of inflammation. Gene expression and secretion of IL-6 and IL-8 in human osteoblasts treated with CoCr (blue dots), TNF (light turquoise dots), or CoCr + TNF (dark blue dots) compared to the untreated control **(A, B)**. The total RNA of osteoblasts was isolated, and relevant genes were examined via qPCR. The results were calculated by the 2^-ΔΔCt^ method and normalized to the unstimulated control. The release of IL-6 **(C)** and IL-8 **(D)** was examined in the supernatants of exposed osteoblasts by ELISA and related to the total protein amount. The results are shown as individual values (one data point per donor) with median and interquartile ranges (n=4). Statistical significance was determined using the 2-way ANOVA and Bonferroni multiple comparison *post hoc* test **(A, B)** and Kruskal-Wallis test with Dunn’s multiple comparisons test **(C, D)**: *p<0.05; ****p<0.0001 (significance between single stimulations); °°p<0.01; °°°°p<0.0001 (significance between CoCr and co-stimulation); ^a^p<0.05; ^b^p<0.01; ^c^p<0.001; ^d^p<0.0001 (significance to unstimulated control). Significant differences within a treatment between time points are listed in the text and not shown in the graphs.

CoCr treatment enhanced *IL6* gene expression between 8 h and 72 h (**Figure 6A**). Over time, expression continued to increase with significant differences at 8 h (p=0.0019) and 24 h (p=0.0436) compared to untreated controls. Osteoblasts incubated with TNF showed upregulated expression after 8 h (p=0.0468), 24 h (p=0.0013), and 72 h (p<0.0001) compared to untreated cells. Treatment of osteoblasts with CoCr + TNF resulted in increased *IL6* mRNA levels after 8 h (p=0.0037), 24 h (p<0.0001), and 72 h (p<0.0001) compared to untreated cells. In Addition, CoCr + TNF and TNF alone led to upregulated *IL6* transcripts after 72 h (p_CoCr+TNF_<0.0001; p_CoCr+TNF_=0.0013) compared to the sole CoCr particle exposure. While treatment with CoCr did not alter IL-6 secretion compared to untreated cells, an increased release was evident over time following treatment with TNF and CoCr + TNF (**Figure 6C**). Here, the release after TNF treatment was significantly increased after 72 h (p=0.0305) compared to untreated osteoblasts.

Osteoblasts treated with CoCr particles for 24 h (p=0.0165) and 72 h (p<0.0001) showed significantly upregulated *interleukin 8* (*CXCL8*) mRNA levels compared to untreated control cells (**Figure 6B**). Further, the expression of *CXCL8* in osteoblasts treated with TNF increased over time (**Figure 6B**). Significantly increased mRNA transcripts were determined after 8 h, 24 h, and 72 h (all; p<0.0001) compared to the control. Compared to the sole CoCr particle exposure, TNF administration led to significantly increased *CXCL8* expression after 8 h, 24 h, and 72 h (p<0.0001). After treatment of osteoblasts with CoCr + TNF, mRNA levels were significantly upregulated from 8 h onwards (all; p<0.0001). The upregulation of *CXCL8* following CoCr + TNF treatment was significant after 8 h, 24 h, and 72 h (all; p<0.0001) compared to the sole CoCr particle exposure. Protein release of IL-8 was increased after treatment with TNF and CoCr + TNF compared to untreated osteoblasts and CoCr exposure alone. After 72 hours of TNF stimulation, a significantly increased IL-8 release was detected compared to the unstimulated control (p=0.0054) and CoCr-treatment (p=0.0216) (**Figure 6D**).

## 4 Discussion

Although it is already known that wear debris can activate the NLRP3 inflammasome of macrophages, which can lead to increased release of IL-1β (28), it is not yet fully understood whether the NLRP3 inflammasome is also primed and activated in bone forming osteoblasts, which come into contact with wear debris at a very early stage and may contribute to the inflammatory process (1, 29). The extent to which wear particles alone can affect the priming of the NLRP3 inflammasome is controversial. In particular, the activation of TLRs by wear particles, known to play a role in aseptic loosening (30, 31), is a subject of discussion. In contrast, TLR agonists like LPS and cytokines such as TNF and IL-1β, known to activate NF-κB, are considered typical inducers of priming. It is also controversial to what extent LPS contributes to the aseptic loosening of joint endoprostheses. It has been shown that LPS that enters the bloodstream tends to localize on foreign surfaces such as joint replacement components or wear particles. In this case, it can then contribute to implant loosening. On the other hand, endotoxin concentrations have only been detected in a few samples collected from the tissue around aseptically loosened joint replacements (32). As macrophages secrete TNF and are present in the implant periphery, it has been assumed as a priming signal in the response of macrophages to wear particles (4).

This study observed increased expression of TLRs after exposure to particles. No differences were found between the increase of *TLR2* mRNA caused by LPS or CoCr particles, while exposure to CoCr resulted in a higher *TLR4* expression than LPS or TNF. Although LPS is known to be a ligand and potent activator of TLRs, our results show that treating osteoblasts with CoCr particles could cause a similar or stronger induction of TLR expression. It is also interesting to note that the combined treatment of the cells did not lead to a strong increase in receptor expression compared to the single treatments. Kikuchi et al. (2001) also reported that treatment of mouse osteoblasts with LPS resulted in increased expression of TLR2 while TLR4 remained unchanged (33). Further work found that LPS and TNF could rapidly induce the *TLR2* gene of macrophages while *TLR4* remained unchanged (34). This is probably because a gene region in the *mTLR2* gene contains several consensus sequences for binding different transcription factors, including two for NF-κB that respond to LPS or cytokine stimulation. In contrast, no NF-κB consensus sequence was found in TLR4 promoters, indicating that the different mRNA induction patterns after stimulation could be explained by the differences in promoter sequences. The fact that this was also observed in osteoblasts suggests that TLR2 inducibility is typical among many cells (33). Although Muthukuru et al. (2020) indicate that osteoblasts have a low constitutive expression of TLR2 and TLR4 and are relatively resistant to upregulation after stimulation with TLR ligands, this study demonstrated increased expression of *TLR2* and *TLR4* following treatment with CoCr particles (35). Resistance to upregulation represents a protective mechanism to prevent tissue damage from an excessive inflammatory response. The increase in TLRs after particle contact indicates a sensitivity of the cells to the unknown signals that disrupt this protective mechanism. Another indication is that an increase in *NF-κB* expression could be shown over time. Further studies should validate the effects of the investigated TLR agonists with inhibitors to verify that the effects of agonists on TLR signaling pathways are indeed reliable. NF-κB is constitutively expressed by cells and located in the cytoplasm until activated. An increase in the basal level of *NF-κB* through increased expression may indicate that the sensitivity of the cells and, therefore, the speed of response to certain stimuli should be increased (36). Induction of the gene was not reflected in the translocation of NF-κB into the nucleus, as the particles did not induce translocation. On the other hand, cellular stimulation with LPS or TNF resulted in a very early translocation of NF-κB to the nucleus, which allowed the transcription of certain proinflammatory mediators to be upregulated (34). These results support the generally accepted scheme that particles alone cannot prime the inflammasome via TLRs because they do not induce NF-κB (4, 28). However, changes in gene expression were observed after CoCr treatment, suggesting the involvement of another, TLR-independent response mechanism. This pathway should be elucidated in future studies. Interestingly, human primary osteoblasts responded faster to TNF treatment than to LPS, whereas macrophages were reported to respond quicker and more strongly to LPS (4, 36). This difference, which was also evident in the results of the gene expression analyses, is likely because macrophages, as immune cells, respond strongly to LPS and must activate themselves to remove foreign bodies. In response to their activation, macrophages secrete, among other molecules, TNF, to which osteoblasts can respond. Since the response of osteoblasts to TNF was higher than to LPS in this study, it is more likely that osteoblasts are more responsive to TNF in their physiological environment. This aspect should be considered more focused in further studies, in which the influence of particle-loaded macrophages on cellular communication with osteoblasts should be addressed.

After TLR activation by ligands, there is a MyD88-dependent induction of NF-κB (30), which upregulates the expression of several proinflammatory cytokines such as *TNFA* and *CXCL8*, but also *IL-6* (6, 37, 38). *MyD88* was more strongly expressed after treatment with particles of the cells than with LPS treatment. Since MyD88 serves as an adaptor for the signaling pathways of TLR and IL-1 receptor family members (39), we assumed that this signaling pathway can be influenced by CoCr particles. This is in line with the results of Pearl et al. (2011), who showed that the response of macrophages to PMMA particles depends on MyD88 as part of the TLR pathway (30). Another indication of pathway activation is the activation of the transcription factor activator protein-1 (AP-1) (40), whose transcriptional expression was also significantly upregulated in our studies after treatment with CoCr particles compared to LPS and untreated controls (**Supplementary Figure S10B**).

Because most cells lack sufficient NLRP3 concentrations and do not constitutively express pro-IL1β, activation of NF-κB, which is the priming signal in inflammasome signaling, leads to upregulation of *NLRP3* and *pro-IL1B* (39, 41). Although translocation of NF-κB was observed after LPS or TNF treatment, no increase in *NLRP3* gene expression was shown. These results contradict studies using MG63 osteoblast-like cells (22), where LPS treatment increased *NLRP3* expression. However, it was shown that NLRP3 is constitutively increased in these cells (42), so an upregulation caused by LPS does not seem unexpected. Taken together, the discrepancy in the results could be due to different cell types, as we used primary human osteoblasts, which may show a different response than tumor-derived cell lines (43). A transcription-independent role of priming signaling has also been reported, with acute LPS priming leading to inflammasome activation without immediate NLRP3 induction (44). TNF alone showed strong donor-dependent differences, which may be because TNF modulates osteoblasts’ function depending on their differentiation stage (45). Interestingly, the combined treatments showed a very similar expression pattern compared to each other, which could be due to the influence of the particles. However, the course is more similar to the TNF treatment alone. CoCr + LPS increases *TNFA* mRNA levels (**Supplementary Figure S11**); the associated increased TNF concentration could lead to a delayed induction of *NLRP3* expression. These results support the suggestion that TNF has a greater effect on inflammasome formation in osteoblasts than in macrophages. While exposure to CoCr led to slightly increased *NLRP3* mRNA levels in osteoblasts over time, macrophages showed an unchanged response to wear particles alone (4). These changes in expression could be related to the material of the particles, as it has already been established that in addition to the particle dose, the particles’ chemical composition can also influence inflammasome activation (4, 21, 46). It has been described that especially molybdenum in particulate (4, 47) but also the ionic (47, 48) form can lead to inflammasome induction in macrophages, while cobalt and chromium have only a minor influence (47). The metallic particles used in this study contained 6% molybdenum, which may have contributed to the response of human osteoblasts. Future studies should investigate the role of particle shape, material, and size on the activation of the NLRP3 inflammasome in osteoblasts.

Upregulation of *NLRP3* results in a more robust inflammatory response but is not required for inflammasome activation (6). In contrast, each treatment in this study resulted in increased expression of *pro-IL1B*. Despite increasing mRNA levels, no protein release into the supernatant was detected by IL-1β ELISAs regardless of osteoblast treatment. Since the LDH assay results did not show increased activity and the ASC staining did not show the formation of ASC specks after the treatments, it can be assumed that there was no activation of the inflammasome by a single stimulus, and thus no cytokine could be released. The results of the LDH assay also suggest that the addition of the activating stimulus ATP could lead to the release of IL-1β, as an increase in LDH activity was shown after ATP activation. Therefore, the investigation of IL-1β release after TNF priming and CoCr priming with an additional activation stimulus should be the focus of future studies. However, the decrease in *pro-IL1B* expression after 8 h suggested that a desensitization process to LPS as a TLR4 agonist may occur to limit the response and protect against damage (10). In addition, different mechanisms of IL-1β release have been described (49). It could be demonstrated that IL-1β has a very short half-life in serum (50). For this reason, it is partially secreted in a protected form via exosomes (51). Protected-released IL-1β would not be detectable via ELISA, so we tried to measure the total intracellular concentration of IL-1β by lysing the cells. In contrast with either TNF, LPS, or combined stimulation, IL-1β levels were not altered after CoCr particle exposure compared to the untreated control. This was unexpected since we detected a significant increase in gene expression after particle exposure. These results suggest that the intensity of the particle stimulus was too low, and only the intracellular storage of IL-1β was detected (49). Therefore, further studies should consider a new stimulation scheme more in line with the pathophysiology of aseptic loosening, where a sequential addition of particles could lead to the induction of IL-1β production. Furthermore, the influence of the particles on release mechanisms and pyroptosis should be further investigated.

Priming signals have been reported not to affect the expression of pro-caspase 1 and other components of the NLRP3 inflammasome (6), and this study also found little effect of the individual treatments on *CASP1* expression. In contrast, the combined stimulations resulted in greater expression of *CASP1*, which fits well with reports that overstimulation of the inflammasome causes to pyroptosis, with increased caspase-1 expression (5). However, the lack of IL-1β release could also be due to a reduced caspase-1 activity, as inhibition of caspase-1 is therapeutically most effective in inhibiting IL-1β release (49, 52). The influence of CoCr particles on caspase-1 should be investigated in further studies.

Treatment of the cells with particles alone or in combination with LPS or TNF appeared to affect gene expression of *CATB*. Phagocytosis of particles, especially large and irregularly shaped particles (9), destabilizes the lysosomal membrane, resulting in the release of cathepsin B (13, 53). The CoCr particles used in this study had a flaky, cauliflower-like shape and an average size of 500 nm (54), making them phagocytizable (21, 55). The uptake of particles after osteoblasts was investigated using hyperspectral darkfield microscopy. The visible shift of the spectral peaks after 0.5 h compared to 10 min indicates phagocytosis of the particles by the cells (56). The decrease in signal intensity during this process indicates that the particles could be surrounded by intracellular structures, e. g. lysosomes, that attenuate the signal from the particles. Since the particles can cause lysosomal damage (57), cathepsin B is consequently released into the cytoplasm. This in turn might induce higher *CATB* mRNA levels following particle exposure to reproduce active cathepsin B. After preparation of the inflammasome, activation occurs through various signals, including the release of cathepsin B (6, 9). However, the influence of cathepsin B on inflammasome activation has not yet been fully elucidated and is therefore the subject of ongoing research (58).

Exposure of cells to particles of various materials can lead to the formation of reactive oxygen species in mitochondria or phagosomes (49, 59). Further, the release of cathepsin B can trigger ROS accumulation within the cell (58). It has been suggested that ROS could lead to the priming of the inflammasome by affecting the NF-κB pathway (10, 60). This raises the question of whether the CoCr particles in this study may not trigger a priming signal directly but rather interact as a secondary effector via the induction of ROS. ROS represent another important activator of the inflammasome. Due to the diverse physical and chemical nature of NLRP3 activators, it is unlikely that NLRP3 physically interacts directly with all known triggers. It is hypothesized that the activators of the NLRP3 inflammasome lead to the induction of ROS, which NLRP3 then senses and activates (61). This study showed that treatment of osteoblasts with CoCr particles alone or in combination resulted in increased ROS formation compared to LPS or TNF treatment alone. Despite these results, it was not possible to detect the activation of the inflammasome. Whether this is due to the lack of a second stimulus as an individual activation signal should be investigated in further studies.

To characterize the inflammatory response of human osteoblasts in response to CoCr particles, IL-6 and IL-8 were investigated as proinflammatory mediators in addition to IL-1β. While the activation and release of IL-1β is controlled by the proteolytic pathway of the inflammasome in addition to transcriptional regulation, the release of IL-8 is independent of inflammasome induction. While all treatments increased the expression of both inflammatory mediators, osteoblasts treated with CoCr alone did not show increased secretion of the proteins. Previous studies from our group found that treatment of osteoblasts with CoCr particles did not induce protein secretion, although mRNA levels were increased (62). The result was explained by the corrosion behavior of the CoCr particles and the associated release of ions that affect cellular mechanisms. It is also possible that the particles affect the protein biosynthesis of osteoblasts by having a cytotoxic effect on cell organelles, which could be triggered by the formation of ROS (56, 63, 64). However, the particle concentration was chosen here because osteoblasts’ viability and morphology are better preserved at 0.01 mg/ml CoCr, as osteoblasts are sensitive to particles (62).

Furthermore, it is controversial whether particles primarily affect IL-8 production or whether the increased IL-8 production is because particles can induce hypoxia, leading to increased IL-8 production. In this regard, our data show that *HIF-1α* gene expression is only induced after 72 h (**Supplementary Figure S10A**). In contrast, increased *CXCL8* levels were already evident after 24 h, suggesting that particles primarily cause the early responses.

The results of the combined stimulations in this study indicate that the timing of the signaling plays a crucial role. The combined stimulations in this study with CoCr + LPS or CoCr + TNF never resulted in significantly higher induction of genes associated with inflammasome signaling than single stimulation, even though both components were present in the medium of the cells. In contrast, other studies have observed a strong induction of IL-1β secretion when cells were pre-treated with LPS or TNF and then stimulated with wear particles. It is also possible that simultaneous treatment of cells with CoCr + LPS or CoCr + TNF resulted in overstimulation, which either induced NLRP3 assembly, which can bypass the priming step and is critical for immediate defense (65), or, more likely, resulted in cell death. In further experiments, the dependence of the cells’ response on the timing of the respective signals should be characterized in detail.

Concluding, in our present study, CoCr particles were shown to influence the expression of NLRP3 inflammasome-associated genes in human osteoblasts from OA patients, although no direct activation of the TLR pathway via translocation of NF-kB was detected. These results suggest the involvement of a TLR-independent response mechanism, which should be elucidated in further studies. A single CoCr stimulus was not sufficient to prime and activate NLRP3. In contrast, TNF appears to be a potent priming signal for human osteoblasts. Interestingly, the effect of TNF on these osteoblasts appeared to be similar to that of LPS, suggesting that these cells are more sensitive to TNF than other phagocytic cells.

## Data availability statement

The original contributions presented in the study are included in the article/**Supplementary Materials**, further inquiries can be directed to the corresponding author/s.

## Ethics statement

The studies involving humans were approved by Ethical Committee of University of Rostock. The studies were conducted in accordance with the local legislation and institutional requirements. The participants provided their written informed consent to participate in this study.

## Author contributions

MS: Conceptualization, Data curation, Formal Analysis, Investigation, Methodology, Validation, Visualization, Writing – original draft, Writing – review & editing. DH: Investigation, Methodology, Writing – review & editing. RB: Funding acquisition, Resources, Writing – review & editing. AJ: Conceptualization, Data curation, Funding acquisition, Methodology, Project administration, Supervision, Writing – review & editing.
