# Supplementary material for: Influence of metallic particles and TNF on the transcriptional regulation of NLRP3 inflammasome-associated genes in human osteoblasts
Source: Front Immunol. 2024 May 1;15:1397432. doi: 10.3389/fimmu.2024.1397432 (PMC11094288; doi:10.3389/fimmu.2024.1397432)
Supplement: Supplementary file 1 [file DataSheet_1.pdf]

Supplementary Material

1 Supplementary Figures

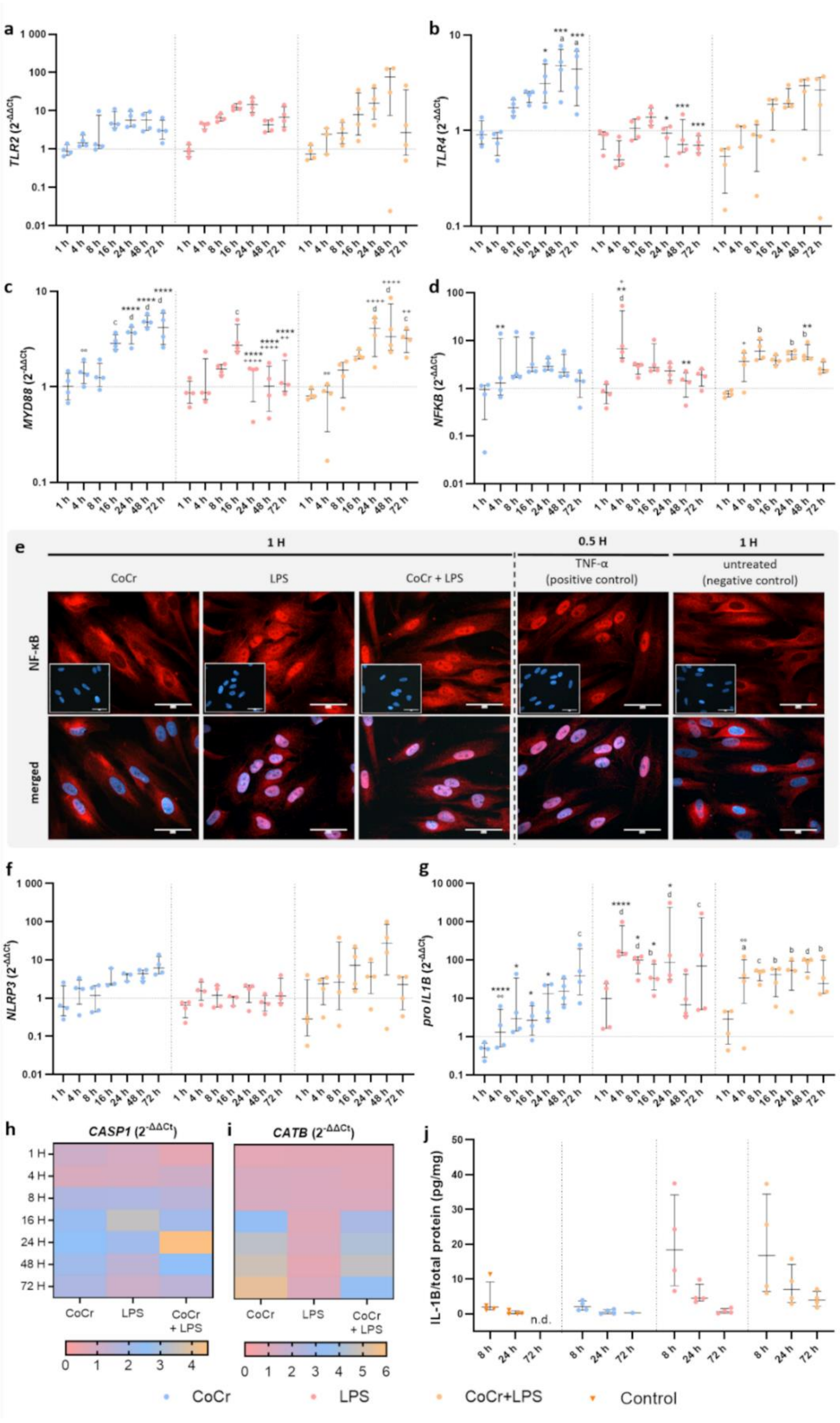

Supplementary Figure S1: Gene expression analysis of markers of TLR signaling (a-d) and NLRP3 inflammasome (f-j) of human osteoblasts treated with CoCr (blue dots), LPS (pink dots), or CoCr + LPS (yellow dots) compared to the untreated control (orange triangle) and evaluation of NFκB translocation to the nucleus (e). (a-d, f-i) The total RNA of osteoblasts was isolated, and relevant genes were examined via qPCR. The results were calculated by the 2-ΔΔCt method and normalized to the unstimulated control (dotted line; in heatmaps: 1). (j) Intracellular IL-1β protein concentration of cell lysates after 8 h, 24 h, and 72 h of stimulation was examined by ELISA and related to the total protein amount. The results were shown as individual values with median and interquartile ranges (a-d, f, g, j) or as median within the heatmaps (h, i; n=4). Statistical significance was determined using the 2-way ANOVA and Bonferroni multiple comparison post hoc test (a-i) and Kruskal-Wallis test with Dunn's multiple comparisons test (j): *p<0.05; **p<0.01; ***p<0.001; ****p<0.0001 (significance between single stimulations); °p<0.05; °°p<0.01; °°°p<0.001; °°°°p<0.0001 (significance between CoCr and CoCr + LPS); $^{+}$p<0.05; $^{++}$p<0.01; $^{+++}$p<0.001; $^{++++}$p<0.0001 (significance between LPS and CoCr + LPS); ^{a}p<0.05; ^{b}p<0.01; ^{c}p<0.001; ^{d}p<0.0001 (significance to unstimulated control).

a

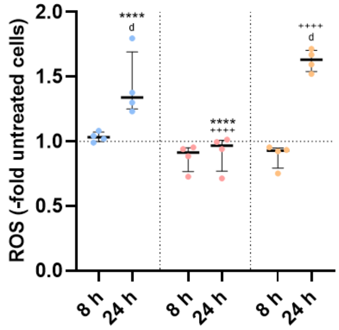

b

	Supernatant (nM)		Lysat (nM)	
	8 h	24 h	8 h	24 h
CoCr	766.75 ± 15.82	247.75 ± 29.73	43.23 ± 1.7	38.33 ± 1.57
LPS	641 ± 32.13	145.25 ± 9.78	44.65 ± 1.72	36.85 ± 2.2
CoCr + LPS	659.25 ± 30.15	287.5 ± 13.90	37.55 ± 3.1	37.25 ± 2.35
untreated	741.75 ± 6.76	167.25 ± 3.82	41.73 ± 6.54	32.375 ± 0.87

Supplementary Figure S2: (a) Total free radical concentration from the supernatant and lysed cells after 8 h and 24 h treatment with CoCr particles (blue dots), LPS (pink dots), or CoCr + LPS (yellow dots) of cells relative to untreated control. Statistical significance was determined using the 2-way ANOVA and Bonferroni multiple comparison post hoc test (a): ****p<0.0001 (significance between single stimulations); $^{++++}$p<0.0001 (significance between LPS and CoCr + LPS); ^{d}p<0.0001 (significance to unstimulated control). Significant differences within a treatment between time points are listed in the text and not shown in the graphs. (b) Free radical concentration in the supernatant and from lysed cells after 8 h and 24 h. Data in the table are presented as mean values ± SEM (n=4).

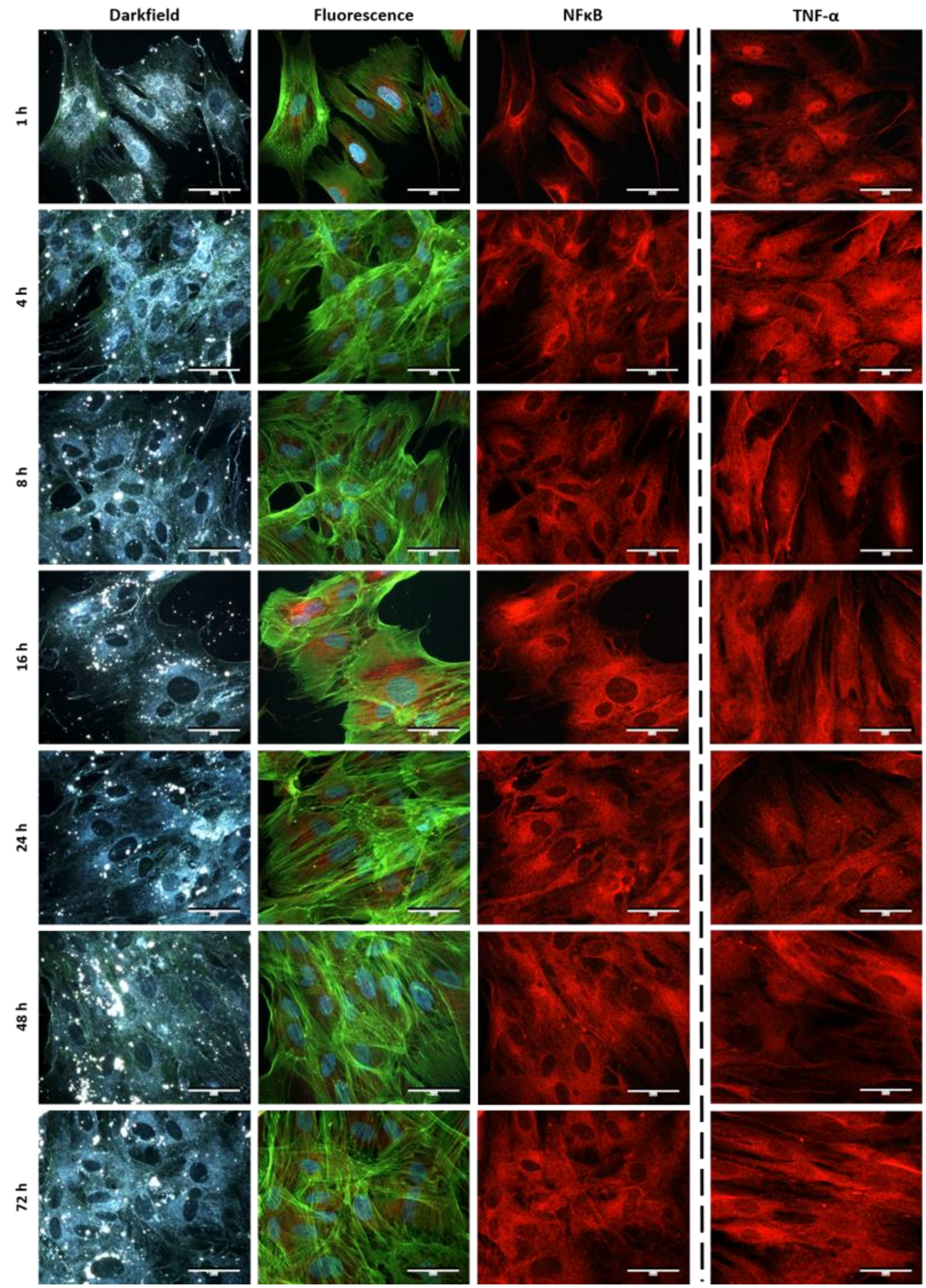

Supplement Figure S3: Evaluation of the translocation of NF-κB to the nucleus of osteoblasts after CoCr particle exposure. Osteoblasts stimulated with TNF-α served as positive controls. Microscopic examinations were performed using the CytoViva® Enhanced Darkfield Hyperspectral Microscope System. The green fluorescent cytoskeleton was imaged at a wavelength of 525 nm. The blue fluorescence of the nuclei stained with DAPI was recorded at a wavelength of 461 nm. The red fluorescence of the NF-κB signal was detected at a wavelength of 625 nm. In addition, images were acquired in the darkfield to visualize unstained structures such as intracellular vesicles, large endosomes, and granules. Bar: 50 µm

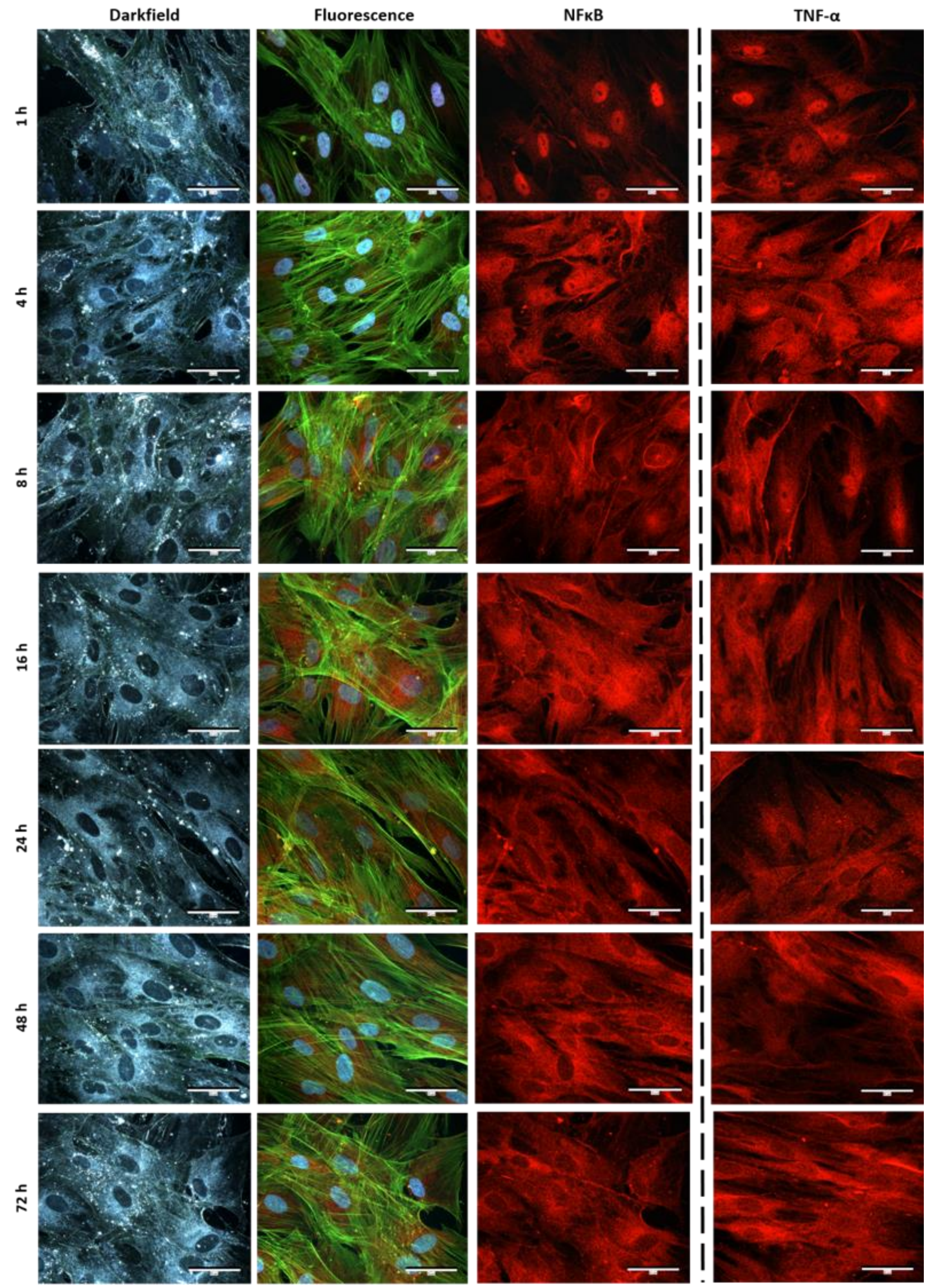

Supplement Figure S4: Evaluation of the translocation of NF-κB to the nucleus of osteoblasts after LPS exposure. Osteoblasts stimulated with TNF-α served as positive controls. Microscopic examinations were performed using the CytoViva® Enhanced Darkfield Hyperspectral Microscope System. The green fluorescent cytoskeleton was imaged at a wavelength of 525 nm. The blue fluorescence of the nuclei stained with DAPI was recorded at a wavelength of 461 nm. The red fluorescence of the NF-κB signal was detected at a wavelength of 625 nm. In addition, images were acquired in the darkfield to visualize unstained structures such as intracellular vesicles, large endosomes, and granules. Bar: 50 µm

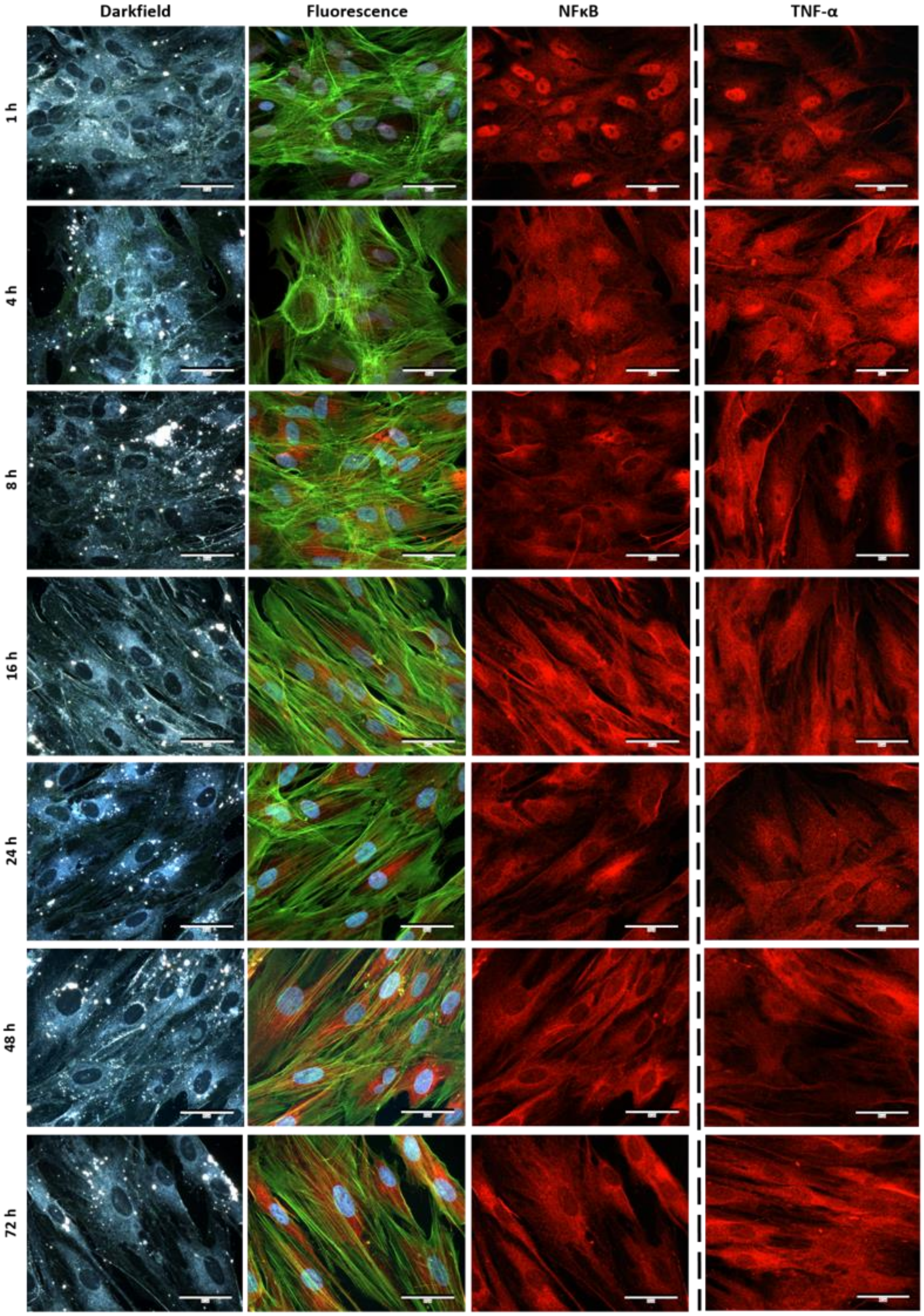

Supplement Figure S5: Evaluation of the translocation of NF-κB to the nucleus of osteoblasts after CoCr + LPS exposure. Osteoblasts stimulated with TNF-α served as positive controls. Microscopic examinations were performed using the CytoViva® Enhanced Darkfield Hyperspectral Microscope System. The green fluorescent cytoskeleton was imaged at a wavelength of 525 nm. The blue fluorescence of the nuclei stained with DAPI was recorded at a wavelength of 461 nm. The red fluorescence of the NF-κB signal was detected at a wavelength of 625 nm. In addition, images were acquired in the darkfield to visualize unstained structures such as intracellular vesicles, large endosomes, and granules. Bar: 50 µm

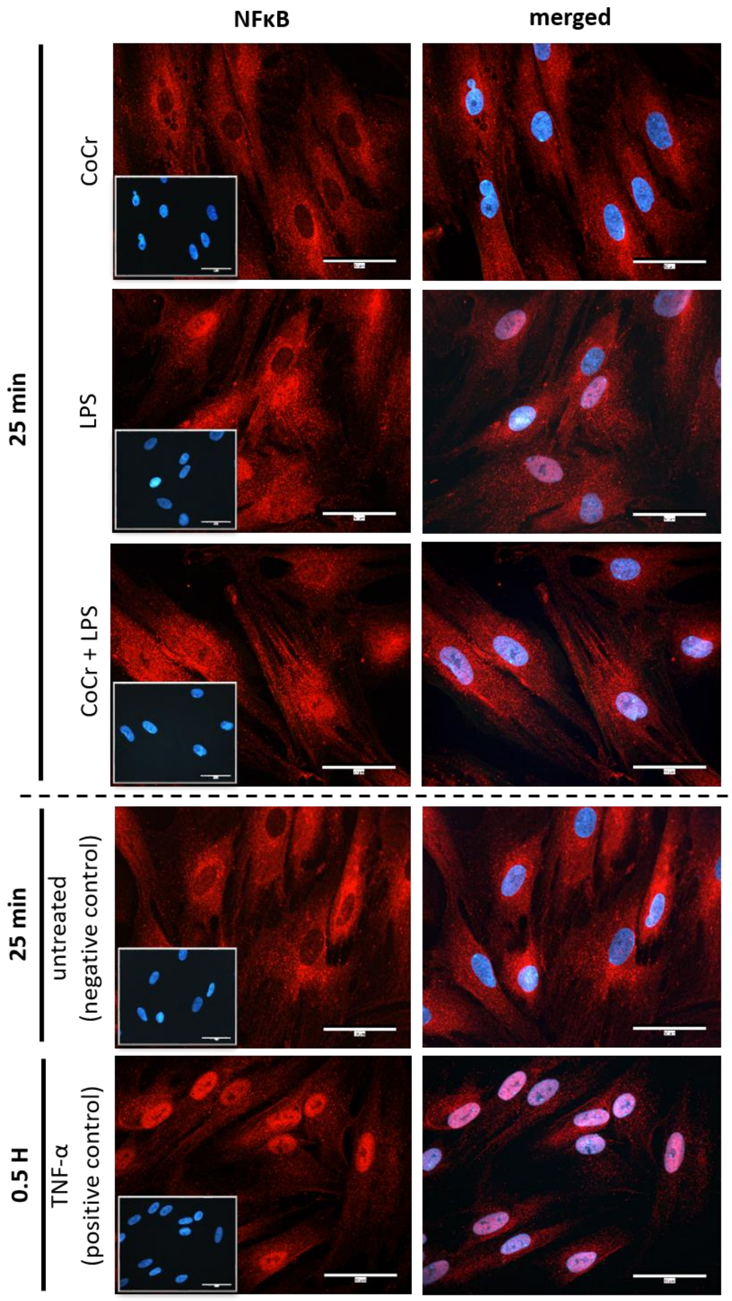

Supplement Figure S6: Evaluation of the translocation of NF-κB to the nucleus of osteoblasts after CoCr, LPS, or CoCr + LPS exposure over 25 min. Osteoblasts stimulated with TNF-α served as positive controls. Microscopic examinations were performed using the CytoViva® Enhanced Darkfield Hyperspectral Microscope System. The blue fluorescence of the nuclei stained with DAPI was recorded at a wavelength of 461 nm. The red fluorescence of the NF-κB signal was detected at a wavelength of 625 nm. Bar: 50 µm

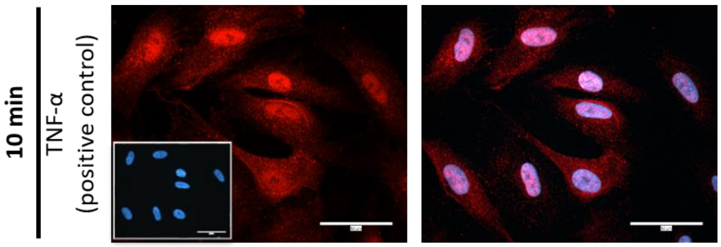

Supplement Figure S7: Evaluation of the translocation of NF-κB to the nucleus of osteoblasts after TNF-α exposure over 10 min. Microscopic examinations were performed using the CytoViva® Enhanced Darkfield Hyperspectral Microscope System. The blue fluorescence of the nuclei stained with DAPI was recorded at a wavelength of 461 nm. The red fluorescence of the NF-κB signal was detected at a wavelength of 625 nm. Bar: 50 µm

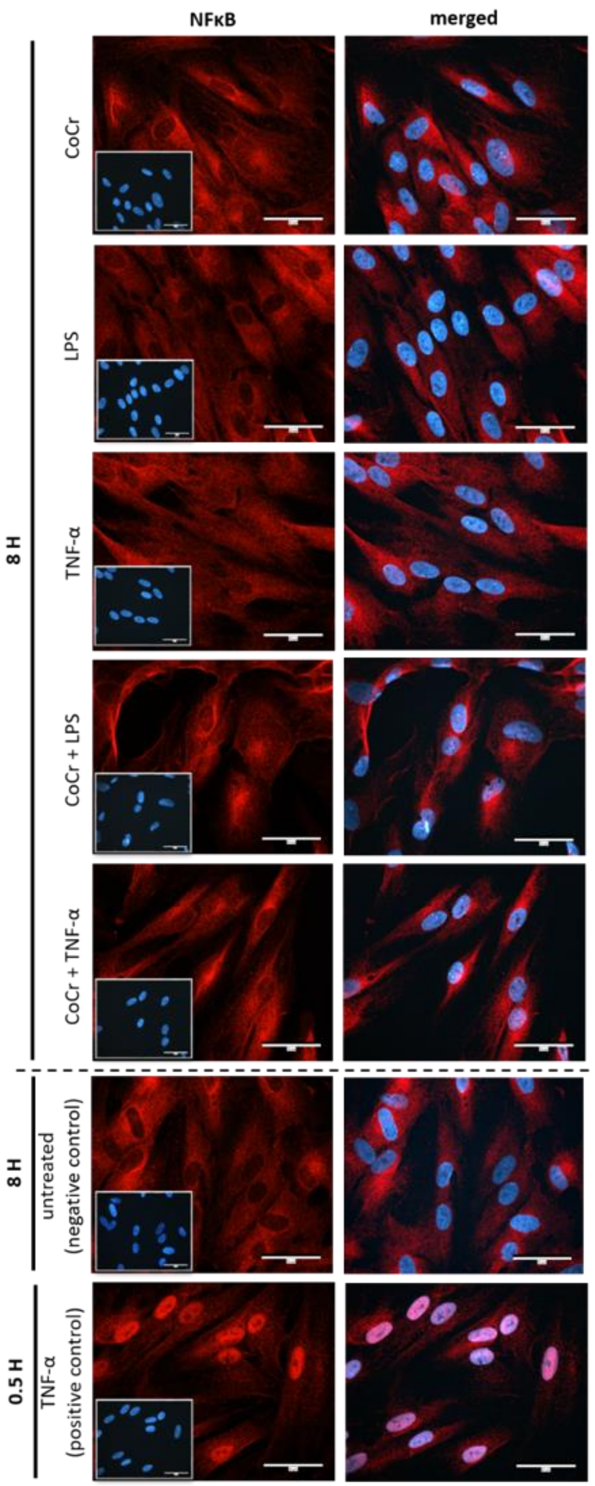

Supplement Figure S8: Evaluation of the translocation of NF-κB to the nucleus of osteoblasts after CoCr, LPS, CoCr + LPS, TNF-α, or CoCr + TNF-α exposure over 8 h. Microscopic examinations were performed using the CytoViva® Enhanced Darkfield Hyperspectral Microscope System. The blue fluorescence of the nuclei stained with DAPI was recorded at a wavelength of 461 nm. The red fluorescence of the NF-κB signal was detected at a wavelength of 625 nm. Bar: 50 µm

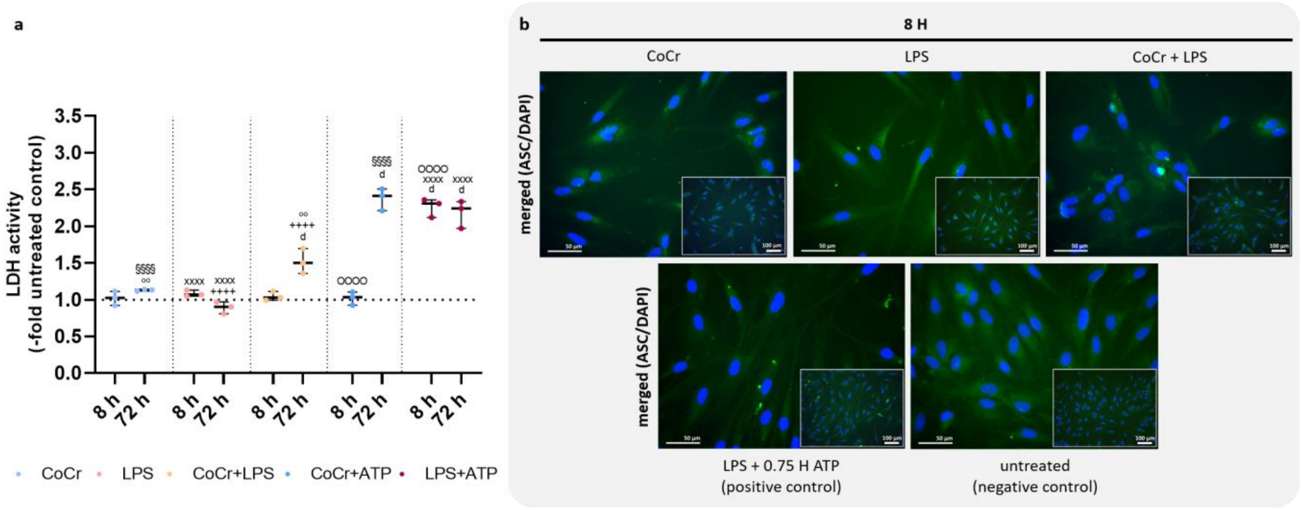

Supplement Figure S9: Effects on lactate dehydrogenase (LDH) activity (a) and staining of ASC-speck formation. LDH activity was measured in supernatants of osteoblasts after exposure with CoCr (blue dots), LPS (pink dots), CoCr+LPS (yellow dots), CoCr+ATP (dark blue dots), and LPS+ATP (red dots). The results are shown as individual values (one data point per donor) with median and interquartile ranges (n=3). Statistical significance was determined using the 2-way ANOVA and Bonferroni multiple comparison post hoc test: $^{++++}p<0.0001$ (significance between LPS and CoCr + LPS); $^{d}p<0.0001$ (significance to unstimulated control); $^{\S\S\S\S}p<0.0001$ (significance between CoCr and CoCr + ATP); $^{XXXX}p<0.0001$ (significance between LPS and LPS + ATP); $^{OOOO}p<0.0001$ (significance between CoCr + ATP and LPS + ATP). An ASC (green) staining was used to investigate the formation of NLRP3 inflammasome after stimulating osteoblasts with CoCr particles, LPS, and CoCr + LPS for 4 h, 8 h, and 24 h. Unstimulated cells were included as negative controls. Osteoblasts treated with LPS and ATP served as positive control. The nuclei were stained with DAPI (blue). Bars: 50 µm; 100 µm

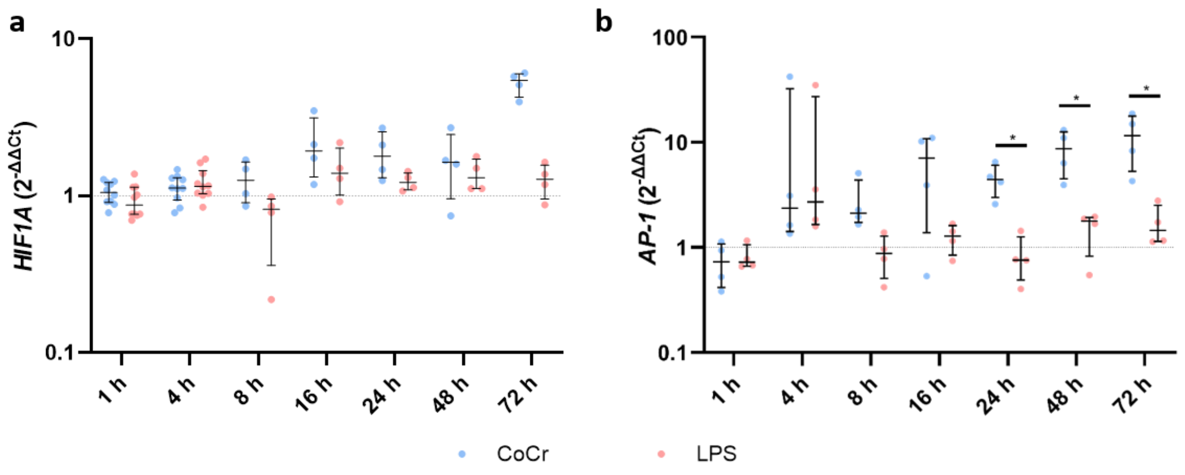

Supplement Figure S10: Analysis of *HIF1-α* (a) and *AP-1* (b) gene expression of human osteoblasts treated with CoCr (blue dots) or LPS (pink dots), compared to the untreated control. The total RNA of osteoblasts was isolated, and relevant genes were examined via qPCR. The results were calculated by the $2^{-\Delta\Delta Ct}$ method and normalized to the unstimulated control (dotted line). The results were shown as individual values with median and interquartile ranges (n=4). Statistical significance was determined using the 2-way ANOVA and Bonferroni multiple comparison post hoc test: *p<0.05 (significance between single stimulations). Significant differences within a treatment between time points are not shown in the graphs.

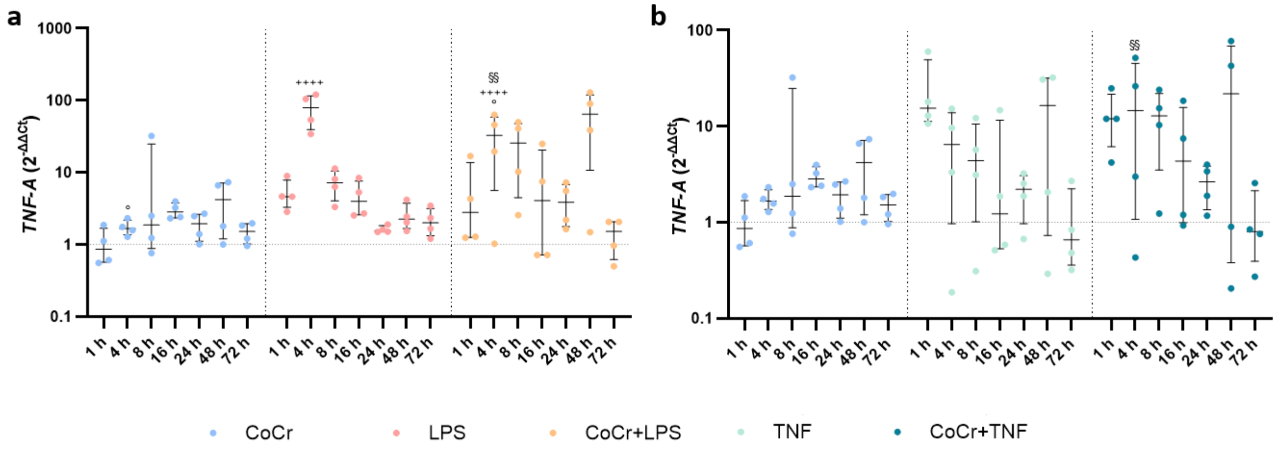

Supplement Figure S11: Analysis of *TNF-α* gene expression of human osteoblasts treated with CoCr (blue dots), LPS (pink dots), CoCr + LPS (yellow dots), TNF-α (light turquoise dots), or CoCr + TNF-α (dark blue dots) compared to the untreated control. The total RNA of osteoblasts was isolated, and relevant genes were examined via qPCR. The results were calculated by the $2^{-\Delta\Delta Ct}$ method and normalized to the unstimulated control (dotted line). The results were shown as individual values with median and interquartile ranges (n=4). Statistical significance was determined using the 2-way ANOVA and Bonferroni multiple comparison post hoc test: °p<0.05 (significance between CoCr and CoCr + LPS); $^{++++}$p<0.0001 (significance between LPS and CoCr + LPS); §§p<0.01 (significance between LPS and TNF-α). Significant differences within a treatment between time points are not shown in the graphs.
